# TREM2 Drives Neutrophil Extracellular Traps‐Induced Dendritic Cell Maturation and Contributes to Lupus Progression

**DOI:** 10.1002/advs.202508938

**Published:** 2025-11-23

**Authors:** Jingxian Shu, Jiabi Liang, Linda Zeng, Shuping Zhong, Xueling Fang, Yating Xu, Yongjian Wu, Xi Huang

**Affiliations:** ^1^ Center for Infection and Immunity, Guangdong Provincial Engineering Research Center of Molecular Imaging The Fifth Affiliated Hospital of Sun Yat‐sen University Zhuhai 519000 China; ^2^ Department of pharmacy The Fifth Affiliated Hospital of Sun Yat‐sen University Zhuhai 519000 China; ^3^ Key Research Laboratory of Traditional Chinese Medicine in the Prevention and Treatment of Infectious Diseases, Traditional Chinese Medicine Bureau of Guangdong Province the Fifth Affiliated Hospital Sun Yat‐Sen University Zhuhai 519000 China; ^4^ Rheumatology and Immunology Department The Fifth Affiliated Hospital of Sun Yat‐sen University Zhuhai 519000 China; ^5^ National Clinical Research Center for Infectious Disease Shenzhen Third People' s Hospital Shenzhen 518112 China

**Keywords:** dendritic cell, lupus nephritis, myeloperoxidase, neutrophil extracellular traps, systemic lupus erythematosus, triggering receptor expressed on myeloid cells 2

## Abstract

Systemic lupus erythematosus (SLE) is a severe autoimmune disease characterized by hyperactive immune cells and excessive autoantigen accumulation. Dendritic cells (DC) can recognize multiple autoantigens and then leading to an inflammatory response, thereby playing a key role in the immunopathogenesis of SLE. However, the regulatory factors underlying DC function remain inadequately clarified. This study identifies that triggering receptor expressed on myeloid cells 2 (TREM2) is upregulated on DCs and is associated with SLE disease severity. Furthermore, TREM2 deficiency in DCs alleviates kidney damage and reduces serum anti‐dsDNA antibody levels, proteinuria, splenomegaly, and lymphadenopathy in lupus mice. Mechanistically, this study demonstrates that TREM2 recognizes neutrophil extracellular traps (NETs) to promote DC maturation and antigen presentation, thereby exacerbating the autoimmune response. More importantly, NETs‐derived myeloperoxidase (MPO) acts as a nonclassical ligand and interacts with TREM2 to activate DAP12/SYK/ERK. Subsequently, TREM2 facilitates NETs uptake by DCs, thereby activating the cGAS/STING signaling pathway. Inhibition of NETs formation or MPO effectively alleviates TREM2‐mediated lupus progression. Collectively, these findings reveal a novel modulatory role of TREM2 and NETs‐derived MPO in the pathogenesis of SLE, which may provide potential options for the treatment of SLE.

## Graphical abstract

TREM2 recognizes NETs‐derived MPO to promote DC maturation and antigen presentation, thereby exacerbating the autoimmune response in SLE. Mechanistically, TREM2 activation triggers the DAP12/SYK/ERK cascade and enhances NETs internalization by DCs, which in turn activates the cGAS/STING signaling pathway. Collectively, this study demonstrates that TREM2 drives SLE pathogenesis by sequentially initiating two key signaling events.

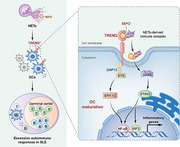

## 1 Introduction

Systemic lupus erythematosus (SLE) is a complex, multifactorial autoimmune disease with severe clinical manifestations.^[^ ^1^ ^]^ The global prevalence of SLE is estimated at 43.7 per 100 000, corresponding to ≈3.41 million affected individuals worldwide.^[^ ^2^ ^]^ SLE is characterized by the accumulation of autoantigens, hyperactive immune cells, aberrant antibody responses, and ultimately, inflammatory organ damage.^[^ ^3^ ^]^ Traditional regimens based on chronic steroids and high‐dose chemotherapeutic agents can alleviate disease progression but often cause severe adverse reactions.^[^ ^4^ ^]^ Thus, it is urgent to develop novel therapeutic targets for SLE.

In SLE pathogenesis, autoantigens derived from apoptotic cells or neutrophil extracellular traps (NETs) are the triggering signals of autoimmunity.^[^ ^5^ ^]^ The impaired clearance increases autoantigen exposure and immune cell activation.^[^ ^3^, ^6^ ^]^ NETs are web‐like DNA structures decorated with nuclear and granule proteins.^[^ ^7^ ^]^ Key molecules externalized in NETs, including myeloperoxidase (MPO), DNA, and LL37 protein, act as autoantigens and immunostimulatory molecules that activate immune responses via the TLR‐dependent pathway.^[^ ^8^, ^9^ ^]^

Subsequently, innate sensing of autoantigens is considered to be the triggering event leading to self‐reactive immunity. Dendritic cells (DCs), as the key antigen‐presenting cells, link innate and adaptive immunity.^[^ ^10^ ^]^ DCs can recognize multiple autoantigens via pattern recognition receptors (PRRs), leading to cell maturation and sustained secretion of inflammatory cytokines.^[^ ^11^, ^12^ ^]^ Mature DCs can further activate the adaptive immune response, promoting the differentiation of T and B cells and the production of pathogenic autoantibodies, which ultimately leads to organ damage.^[^ ^13^ ^]^ Therefore, identifying the autoantigens and critical molecules that regulate DC maturation is essential for developing novel SLE therapies.

A series of cytoplasmic PRRs in myeloid cells, such as TLRs, NLRPs, and cGAS, are responsible for detecting pathogenic antigens, promoting cell maturation.^[^ ^14^, ^15^, ^16^ ^]^ Several regulators have been reported to modulate TLR signaling pathways, such as triggering receptors expressed on myeloid cells (TREMs). TREMs belong to a family of cell surface receptors that are broadly expressed on myeloid cells, including DCs, macrophages, and monocytes.^[^ ^17^, ^18^ ^]^ For instance, TREM1 expressed on DCs suppresses TLR9‐induced inflammation response in lupus mice.^[^ ^19^ ^]^ TREM2 has been reported to be involved in the regulation of inflammation, cell survival, proliferation, and phagocytosis.^[^ ^20^ ^]^ It binds to diverse ligands, including phospholipids, lipoproteins, proteins, and apoptotic cells, transducing intracellular signals via the adaptor protein DAP12.^[^ ^20^ ^]^ In recent years, the regulatory functions of TREM2 in DCs have gained increasing attention. In vitro studies indicate that TREM2 can promote DC maturation and survival,^[^ ^21^ ^]^ and it exacerbates mucosal inflammation in murine colitis by enhancing the inflammatory response of DCs.^[^ ^22^ ^]^ However, the role of TREM2 in DC regulation appears context‐dependent. For example, in a model of liver ischemia‐reperfusion injury, TREM2 negatively regulates DCs' maturation and immunostimulatory functions.^[^ ^23^ ^]^ These conflicting findings highlight the complexity of TREM2 in DC biology. Nevertheless, how the autoantigen–TREM2–DC axis operates in SLE remains incompletely understood.

In this study, we demonstrated that TREM2 expressed on DCs plays a key role in aggravating lupus progression. The knockout of TREM2 in DCs alleviated the symptoms and organ damage in lupus mice. Mechanistically, we clarified that TREM2 promotes DC maturation and phagocytosis by interacting with NETs‐derived MPO. Taken together, our study provides molecular and cellular insights into the manipulation of lupus progression, and proposes that combined TREM2 blockade and inhibition of NETs‐derived MPO represents a promising therapeutic strategy for lupus.

## 2 Results

### 2.1 TREM2 Expression on DCs is Upregulated and Correlated with Disease Severity in SLE

**Figure 1:**
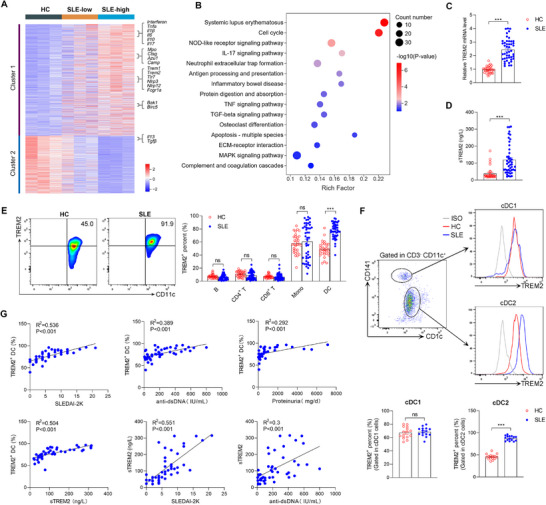
TREM2 expression is upregulated on DCs and correlated with disease severity in SLE patients. A,B) RNA sequencing of healthy controls (HCs, n=3), SLE patients with SLEDAI‐2K scores < 10 (n=3) and SLE patients with SLEDAI‐2K scores ≥ 10 (n=3) were performed. (A) Heatmap of altered genes related to immune system was shown. (B) KEGG pathway analysis was conducted to enrich the differentially expressed genes between HCs and SLE patients. C) The mRNA levels of TREM2 in PBMCs from HCs (n=30) or SLE patients (n=46) were detected by RT‐PCR (unpaired 2‐tailed Student's *t*‐test). D) The plasma sTREM2 levels in HCs (n=30) and SLE patients (n=46) were detected by ELISA (unpaired 2‐tailed Student's *t*‐test). E) The expression levels of TREM2 in CD19^+^ B cells, CD4^+^ T cells, CD8^+^ T cells, CD14^+^ monocytes (Mo), and CD11c^+^ DC from HCs (n=30) and SLE patients (n=46) were analyzed by flow cytometry (unpaired 2‐tailed Student's *t*‐test). F) Flow cytometry was used to detect the expression levels of TREM2 in cDC1 (CD11c^+^ CD141^+^ CD1c^−^) and cDC2 (CD11c^+^ CD141^−^ CD1c^+^) from HCs (n=15) and SLE patients (n=15). The unpaired, 2‐tailed Student's *t*‐test was used to analyze the data. G) Pearson's correlation analysis in SLE patients assessed the relationship of peripheral TREM2^+^ DC percentage with SLEDAI‐2K scores (n=46), serum anti‐dsDNA Abs (n=46), sTREM2 (n=46), proteinuria levels (n=38); and of sTREM2 with SLEDAI‐2K scores (n=46) and serum anti‐dsDNA (n=46). Data are represented as mean ± SEM from at least three independent experiments. ^*^ *P* < 0.05, ^**^ *P* < 0.01, ^***^ *P* < 0.001.

SLE patients who met the 2019 EULAR/ACR classification criteria were enrolled in this study. SLE patients with active infection, malignancy, and other autoimmune diseases were excluded from this study. To explore critical regulatory genes in SLE, RNA sequencing was performed on whole blood cells of SLE patients and healthy controls (HCs). According to the SLE Disease Activity Index 2000 (SLEDAI‐2K) scores, SLE patients were classified into the SLE‐low group (scores < 10) and the SLE‐high group (scores ≥ 10). Cluster analysis showed an upregulation of inflammation‐related genes in the SLE‐high group, including genes encoding inflammatory cytokines (*Interferon, Tnfα, Il1β, Il6, Il10, Il17*) and immune receptors (*Trem1, Trem2, Tlr7, Nlrp3, Nlrp12, Fcgr1a*), genes related to NETs formation (*Mpo, Ctsg, Azu1, Camp*), while genes encoding anti‐inflammatory factors such as *Il13, Tgfβ* were down‐regulated (**Figure** **1**A). In addition, we performed the KEGG pathway enrichment analysis using the differentially expressed genes (DEGs) between SLE patients and healthy controls. The results revealed that DEGs were mainly enriched in SLE, immune system (NOD‐like receptor, IL‐17), NETs formation, antigen processing and presentation signaling‐related pathways (Figure 1B). In the immunopathogenesis of SLE, the triggering event is that myeloid cells recognize antigens via surface or intracellular receptors.^[^ ^10^ ^]^ Among these immune receptors, we observed a 2.5 fold upregulation in the mRNA level of TREM2, a receptor constitutively expressed on myeloid cells, in peripheral blood mononuclear cells (PBMCs) of SLE patients compared with HCs (Figure 1C; Table S1, Supporting Information). In addition, we also observed elevated sTREM2 levels in the plasma of SLE patients (Figure 1D).

Flow cytometry further analyzed TREM2 expression on the surface of the indicated PBMCs subsets from healthy controls and SLE patients. In HCs, TREM2 was mainly expressed on CD14^+^ monocytes and DCs, but not on plasmacytoid DCs (pDCs), CD4^+^ T cells, CD8^+^ T cells, and B cells (Figures S1 and S2, Supporting Information). TREM2 expression was increased exclusively on DCs from SLE patients, while remaining unchanged on other immune cell subtypes (Figure 1E). The conventional DCs can be further divided into CD141^+^ cDC1 and CD1c^+^ cDC2 subpopulations.^[^ ^11^ ^]^ Further analysis of the expression profile across DC subsets revealed a 1.9 fold upregulation of TREM2 expression in the cDC2 subpopulation of SLE patients compared to healthy controls, whereas its expression in the CD141⁺ cDC1 subset showed no significant change (Figure 1F). Subsequently, we analyzed the correlations between TREM2 expression and clinical markers related to disease activity or severity among SLE patients. The percentage of peripheral TREM2^+^ DCs was positively correlated with SLEDAI‐2K scores (R^2^═0.536), the levels of serum anti‐dsDNA Abs(R^2^═0.389), sTREM2 (R^2^═0.504), and proteinuria (R^2^═0.292) (Figure 1G). Similarly, sTREM2 levels, which were elevated in SLE and associated with TREM2⁺ DC frequency, also showed positive correlations with SLEDAI‐2K scores (R^2^═0.551) and anti‐dsDNA antibody levels (R^2^═0.3) (Figure 1G). Therefore, the percentage of peripheral TREM2^+^ DCs and sTREM2 levels represents promising surrogate biomarkers for SLE disease severity. To validate these observations in vivo murine model, we detected the TREM2 expression in lupus mice. Consistent with the above results in SLE patients, DCs from blood, spleen, and lymph node in lupus mice all exhibited elevated TREM2 expression compared with control mice (Figure S3, Supporting Information). Taken together, we observed an increased expression of TREM2 on DCs in both SLE patients and mice, which is positively correlated with disease severity.

### 2.2 TREM2 Deficiency in DCs Alleviates Lupus‐related Manifestations

**Figure 2:**
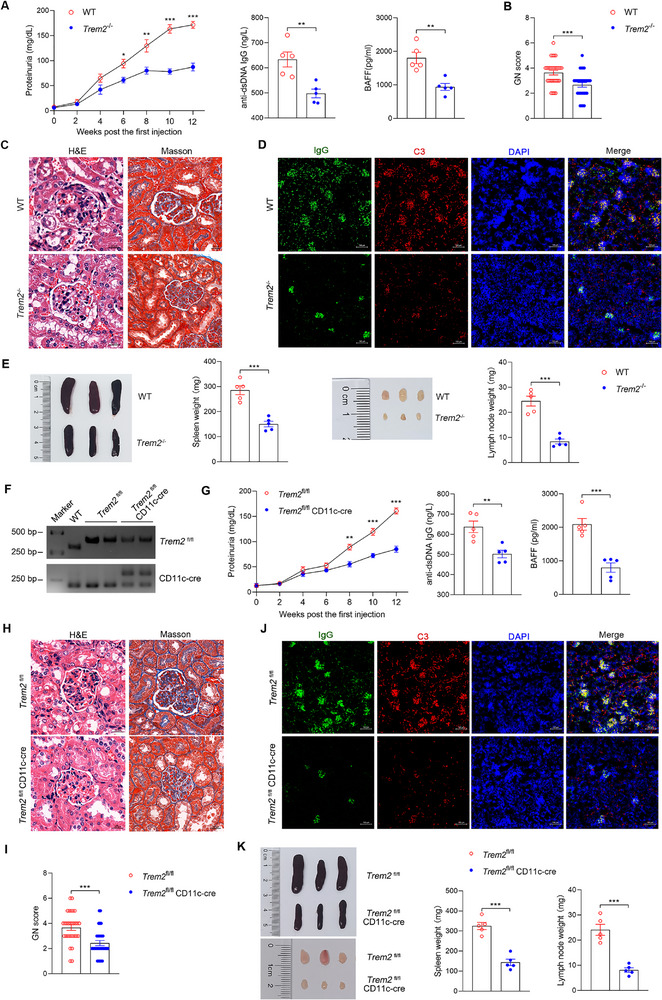
*Trem2* deficiency in DCs alleviates lupus‐related syndrome and organ damage. A–E) ALD‐DNA induced lupus model were established in WT and *Trem2* ^−/−^ mice. The mice were subcutaneously immunized with ALD‐DNA at 0, 2, 4 weeks. (A) Urine samples were collected from 0 to 12 week and proteinuria at each indicated timepoints were detected by ELISA. The serum from each mouse was collected at week 12, and then serum anti‐dsDNA Abs and B‐cell activating factor (BAFF) were detected by ELISA (n=5, unpaired 2‐tailed Student's *t*‐test). (B,C) The paraffin‐embedded kidney sections were stained with H&E and Masson. Scale bar, 20 µm. Kidney pathology was evaluated with glomerulonephritis (GN) scores (n=30, unpaired 2‐tailed Student's *t*‐test). (D) Immunofluorescence assays were used to analyze the deposition of IgG and C3 in renal sections. Scale bar, 100 µm. (E) The gross appearance and the weight of spleens or lymph nodes were recorded at week 12 (n=5, unpaired 2‐tailed Student's *t*‐test). F) *Trem2* ^fl/fl^ mice and *Trem2* ^fl/fl^ CD11c‐cre mice were constructed, and identified by PCR. G–K) ALD‐DNA induced lupus model were established in *Trem2* ^fl/fl^ and *Trem2* ^fl/fl^ CD11c‐cre mice. (G) The levels of proteinuria at each indicated timepoints, serum anti‐dsDNA Ab and BAFF at week 12 were detected by ELISA (n=5, unpaired 2‐tailed Student's *t*‐test). (H,I) Renal damage was evaluated by H&E and Masson staining at week 12. Scale bars, 50 µm (n=30, unpaired 2‐tailed Student's *t*‐test). (J) The deposition of IgG and C3 in renal sections from indicated genotype mice was analyzed by immunofluorescence assays. Scale bar, 100 µm. (K) The appearance and the weight of spleens or lymph nodes were recorded (n=5, unpaired 2‐tailed Student's *t*‐test). The results are presented as the mean ± SEM from at least three separate experiments. ^*^P < 0.05, ^**^P < 0.01, ^***^P < 0.001.

To explore the role of TREM2 in lupus, we employed a nucleic acid antigen‐driven model using activated lymphocyte‐derived DNA (ALD‐DNA)^[^ ^24^ ^]^ to immunize *Trem2* knockout (*Trem2* ^−/−^) and wild type (WT) mice. Within 1–2 months, mice with ALD‐DNA immunization produced high levels of anti‐dsDNA Abs and developed SLE‐like syndrome.^[^ ^25^ ^]^ Results showed that *Trem2* knockout led to 49% and 21% reductions in proteinuria and anti‐dsDNA Abs levels, respectively, in the lupus model at 12 weeks (**Figure** **2**A). B‐cell activating factor (BAFF), secreted by myeloid cells, could promote B‐cell survival and differentiation, which results in the production of autoantibodies and contributes to SLE progression.^[^ ^26^ ^]^ The serum BAFF levels were also decreased in *Trem2* ^−/−^ mice compared with WT mice, with a reduction of ≈48% (Figure 2A). Since glomerulonephritis (GN) and kidney deposition of IgG and complement C3 are the critical features of lupus, histological staining and immunofluorescence assays were used to evaluate the renal damage. H&E and Masson staining showed that *Trem2* ^−/−^ lupus mice displayed marked amelioration in renal damage for the GN scores (Figure 2B, C). Similarly, the deposition of IgG and C3 in kidneys was less pronounced in *Trem2* ^−/−^ lupus mice than in WT lupus mice (Figure 2D). In addition, *Trem2* ^−/−^ lupus mice exhibited significant reductions in the weight of their lymphatic organs, with spleen and lymph node weights ≈48% and 65% lower, respectively, than those of control WT lupus mice (Figure 2E). Together, these results demonstrate that TREM2 deficiency protects against lupus pathogenesis in an antigen‐driven model. To validate the pathogenic role of TREM2 in a distinct, chronic inflammation‐driven context, we employed a pristane‐induced lupus model ^[^ ^27^ ^]^. Consistent with our findings in the ALD‐DNA model, *Trem2* ^−/−^ mice in the pristane model showed significant mitigation of disease severity, as reflected by reduced proteinuria, anti‐dsDNA Abs, and BAFF levels, as well as improved renal and lymphoid organ pathology (Figure S4A–C, Supporting Information). This replication across two mechanistically distinct induced models underscores a fundamental role for TREM2 in lupus pathogenesis.

Subsequently, to assess the therapeutic relevance of TREM2 in a spontaneous and genetically determined setting that closely mirrors human SLE, we utilized lupus‐prone MRL/Lpr mice ^[^ ^28^ ^]^. The MRL/Lpr mice were divided into three groups, which were respectively treated with TREM2 blocking Abs (anti‐TREM2), cyclophosphamide (CTX), and control IgG Abs (Figure S5A, Supporting Information). Notably, pharmacological blockade of TREM2 significantly ameliorated disease progression. The treatment reduced serum anti‐dsDNA antibodies, proteinuria, lupus‐like facial skin lesions, renal damage, and lymphoid organ pathology compared to the control IgG group (Figure S5B–F, Supporting Information). This confirms TREM2 as a promising therapeutic target for intervention in lupus.

Having established TREM2 as a key aggravating factor in lupus through multiple lupus models, we next explore the key cellular mediator. Since we observed upregulated TREM2 expression on DCs during lupus (Figure 1), we generated *Trem2* conditional knockout mice (*Trem2* ^fl/fl^ CD11c‐cre), in which *Trem2* was specifically deleted in DCs (Figure 2F), to explore whether TREM2 exerts a function in lupus via DCs. *Trem2* ^fl/fl^ CD11c‐cre lupus mice displayed reduced levels of proteinuria, anti‐dsDNA Abs, and BAFF compared with control *Trem2* ^fl/fl^ lupus mice (Figure 2G). Simultaneously, *Trem2* ^fl/fl^ CD11c‐cre mice showed alleviated renal damage and less deposition of IgG and C3 (Figure 2H–J). Moreover, the specific deletion of *Trem2* in DCs reduced spleen and lymph node weight by ≈55% and 66%, respectively (Figure 2K). These data indicate that TREM2 expressed on DCs plays a critical role in driving lupus progression. To further elucidate how TREM2 modulates DC function, we performed flow cytometric analysis. While the overall proportion of splenic DCs was unaltered (Figure S6A, Supporting Information), DCs from *Trem2* ^fl/fl^ CD11c‐cre lupus mice displayed significantly reduced expression of maturation markers, including MHC II, CD86, and CCR7 (Figure S6B, Supporting Information). Furthermore, DC‐specific *Trem2* deletion led to a decrease in splenic neutrophils but did not affect the macrophage population (Figure S6C, Supporting Information), indicating a selective impact on myeloid subsets. We subsequently investigated the downstream effects of DC‐specific *Trem2* deletion on the adaptive immune response. B cells, CD4^+^ T cells, follicular T helper cells (TFH cells), Th2 cells, Th17 cells, plasma cells, germinal center B cells (GCB cells), and memory B cells were all decreased in *Trem2* ^fl/fl^ CD11c‐cre lupus mice compared to those in *Trem2* ^fl/fl^ lupus mice (Figure S7A–C, Supporting Information). Conversely, the inactivation marker CD62L of TFH, the percentages of Treg cells, and naïve B cells were higher in *Trem2* ^fl/fl^ CD11c‐cre lupus mice compared with control lupus mice (Figure S7A–C, Supporting Information). The ratios of CD8^+^ T and Th1 cells showed no significant differences between *Trem2* ^fl/fl^ CD11c‐cre and *Trem2* ^fl/fl^ groups (Figure S7A, B, Supporting Information). Collectively, these data indicated that *Trem2* deficiency in DCs restrained their maturation and subsequently dampened the inflammatory response across multiple innate and adaptive immune cell populations, ultimately alleviating lupus pathogenesis.

To further confirm the DC‐mediated role of TREM2 in lupus, we transferred ALD‐DNA‐pretreated WT or *Trem2* ^−/−^ BMDCs (CD45.2⁺) into CD45.1⁺ mice (Figure S8A, Supporting Information).^[^ ^29^, ^30^ ^]^ Two weeks post‐transfer, WT and *Trem2* ^−/−^ BMDCs can also be detected in recipient spleens and lymph nodes (Figure S8B,C, Supporting Information). By week 20, mice receiving *Trem2* ^−/−^ BMDCs exhibited lower anti‐dsDNA antibody levels, reduced proteinuria, milder lupus nephritis, decreased renal IgG and C3 deposition, along with reduced spleen and lymph node weights compared to those receiving WT BMDCs (Figure S8D,J, Supporting Information). These data demonstrated that DCs with *Trem2* deficiency alleviated lupus‐related manifestations. Taken together, we explored the in vivo role of TREM2 in lupus by multiple approaches and revealed that TREM2 aggravated lupus progression via DCs.

### 2.3 NETs are Upregulated and Positively Correlated with TREM2 expression in SLE

**Figure 3:**
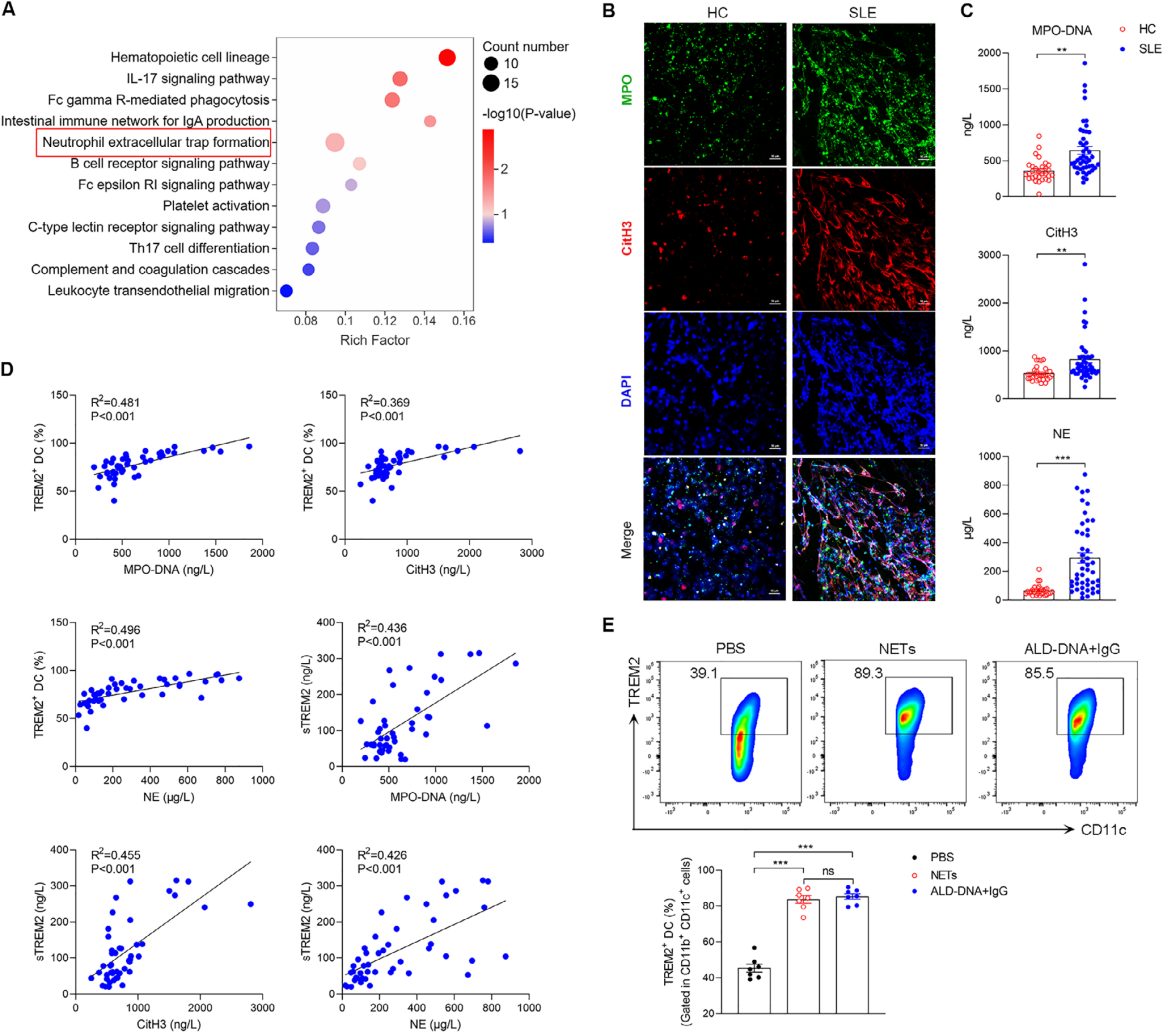
NETs are upregulated and positively correlated with TREM2 expression in SLE. A) The differentially expressed genes between SLE patients with TREM2 high and low expression were conducted to the KEGG enrichment analysis. B) Representative immunostaining fluorescence images for DNA (DAPI, blue), MPO (green) and CitH3 (red) in HC neutrophils, SLE neutrophils after stimulation with anti‐RNP IgG for 4 h. Scale bar, 50 µm. C) ELISA was used to detect the levels of plasma MPO‐DNA complex, CitH3 and NE protein in healthy controls (n=30), SLE patients (n=46). The unpaired, 2‐tailed Student's *t*‐test was used to analyze the data. D) Pearson's correlation analysis in SLE patients assessed the relationship of peripheral TREM2^+^ DC percentage with plasma MPO‐DNA complex, CitH3 protein, NE protein levels (n=46); and of sTREM2 levels with plasma MPO‐DNA complex, CitH3 protein, NE protein levels (n=46). E) TREM2 expression in BMDC (marked in CD11b^+^ CD11c^+^) stimulated with PBS, NETs or DNA+IgG (ICs) were detected by flow cytometry (n=7, One‐way ANOVA). The results are presented as the mean ± SEM from at least three separate experiments. ns, no significance; ^*^ *P* < 0.05; ^**^ *P* < 0.01; ^***^ *P* < 0.001.

In the immunopathological process of SLE, autoantigens derived from apoptotic cells or NETs are considered the triggering signals of autoimmunity.^[^ ^5^ ^]^ Thus, the KEGG analysis was conducted to explore the specific autoantigen associated with TREM2 expression. Based on the RNA‐seq data, we found that the DEGs between SLE patients with TREM2 high and low expression were enriched in NETs formation (**Figure** **3**A). NETs mainly originate from dead neutrophils via NETosis in pathological conditions.^[^ ^8^ ^]^ First, to elucidate the involvement of NETs in SLE, we isolated neutrophils from HCs and SLE patients, and then cultured neutrophils with anti‐RNP IgG for 4 h. The neutrophils from SLE patients, but not healthy controls, underwent NETosis, releasing NETs with the typical web‐like structure (Figure 3B). Next, we detected the plasma levels of MPO‐DNA, citrullinated histone H3 (CitH3), and neutrophil elastase (NE), which are representative indicators of NETs. Compared with healthy controls, SLE patients showed increased MPO‐DNA, CitH3, and NE levels (Figure 3C). Furthermore, we conducted a linear regression analysis to reveal the relationship between TREM2 expression and NETs. The percentage of peripheral TREM2^+^ DC was positively correlated with the plasma MPO‐DNA (R^2^═0.481), CitH3 (R^2^═0.369), and NE (R^2^═0.496) levels in SLE patients (Figure 3D). Moreover, positive correlations were also observed between plasma sTREM2 levels and markers of NETs (Figure 3D). To further investigate the association between NETs and TREM2 expression on DCs, we cocultured BMDCs with NETs or immune complexes (ICs). The results showed that TREM2 expression was upregulated in BMDCs treated with NETs or ICs compared with those treated with PBS (Figure 3E). Together, these data suggested that the NETs showed a positive correlation with TREM2 expressed on DCs.

### 2.4 TREM2 is Essential for NETs‐induced DCs Maturation and Antigen Presentation

**Figure 4:**
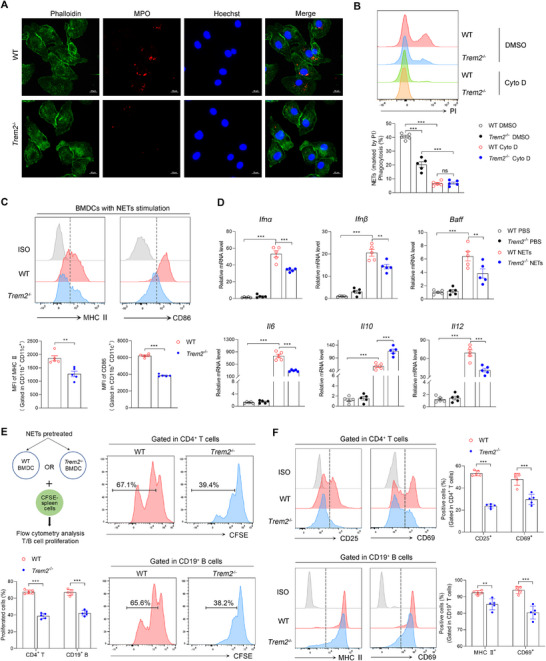
TREM2 drives NETs‐induced DC maturation and antigen presentation via phagocytosis. A) Representative fluorescence images showed that WT and *Trem2* ^−/−^ BMDCs (labeled with green phalloidin) internalized MPO from NETs at 3 h after coculture. MPO was labeled with anti‐MPO rabbit antibody and AF594 conjugated goat anti‐rabbit IgG. The phagocytosis was recorded by confocal microscopy. Scale bar, 10 µm. B) WT and *Trem2* ^−/−^ BMDCs were pretreated with Cytochalasin D (Cyto D) or DMSO for 30 mins. And then BMDCs were co‐cultured with PI‐labeled NETs for 3 h. The phagocytosis was determined by flow cytometry (n=5, one‐way ANOVA). C) WT and *Trem2* ^−/−^ BMDCs were stimulated with NETs for 24 h. The maturation markers (MHC II, CD86) of BMDCs were analyzed by flow cytometry (n=5, unpaired 2‐tailed Student's *t*‐test). D) The cytokines, *Ifnα, Ifnβ, Baff, Il6, Il10 and Il12*, were measured by RT‐PCR (n=5, one‐way ANOVA). E) WT or *Trem2* ^−/−^ BMDCs were pretreated with NETs for 24 h and then were collected for coculture with CFSE‐labeled spleen cells for 3 days. Proliferation of CD4^+^ T cells (CD3^+^ CD4^+^) and B cells (CD3^−^ CD19^+^) was assessed by flow cytometry based on CFSE dilution (n=5, unpaired 2‐tailed Student's *t*‐test). F) The expression of activation markers on CD4^+^ T cells (CD25 and CD69) and B cells (MHC II and CD69) was analyzed by flow cytometry after coculture (n=5, unpaired 2‐tailed Student's *t*‐test). The results are presented as the mean ± SEM from at least three independent experiments. ns, no significance; ^*^ *P* < 0.05; ^**^ *P* < 0.01; ^***^ *P* < 0.001.

Since autoantigen NETs were identified to be associated with TREM2 expressed on DCs, we next explored whether TREM2 mediates the effect of NETs on DCs. Given that TREM2 is known to promote the phagocytosis of various ligands, including apoptotic cells and lipoproteins,^[^ ^20^ ^]^ we speculated that TREM2 also facilitates NETs uptake by DCs. To assess the effect on phagocytosis, we treated WT or *Trem2* ^−/−^ BMDCs with NETs for 3 h. Immunofluorescence analysis showed that *Trem2* deficiency impaired phagocytosis of NETs in BMDCs (**Figure** **4**A). Next, we used the actin polymerization inhibitor cytochalasin D (Cyto D) to confirm that NETs uptake was mediated by active phagocytosis. Cyto D pretreatment completely abolished NETs internalization in both WT and *Trem2* ^−/−^ BMDCs, whereas vehicle DMSO‐pretreated WT BMDCs exhibited significantly greater phagocytic capacity than their *Trem2* ^−/−^ BMDCs (Figure 4B). These results indicated that TREM2 promoted the phagocytosis of NETs by DCs. Subsequently, we investigated the maturation and inflammatory phenotype of DCs following NETs internalization. The flow cytometry analysis showed that *Trem2* deficiency impaired the maturation phenotype (MHC II, CD86) in BMDCs compared to the WT group (Figure 4C; Figure S9, Supporting Information). The mRNA levels of inflammatory cytokines were also detected in DCs. Cytokines such as *Ifnα, Ifnβ, Baff, Il6, Il10*, and *Il12* were more highly expressed in BMDCs with NETs stimulation compared to those with PBS treatment. *Trem2* deficiency reduced the expression of proinflammatory cytokines *Ifnα, Ifnβ, Baff, Il6*, and *Il12*, while the anti‐inflammatory cytokine *Il10* showed the opposite changes and expressed more in *Trem2* ^−/−^ BMDCs treated with NETs than WT BMDCs treated with NETs (Figure 4D).

The mature DCs present antigens to T cells and secrete cytokines to B cells, which can further activate self‐reactive T and B cells underlying SLE pathogenesis.^[^ ^11^ ^]^ To explore whether TREM2 affects antigen‐presentation ability of DCs, we analyzed the proliferation and activation of T cells and B cells in the coculture system. WT and *Trem2* ^−/−^ BMDCs were pretreated with NETs, followed by in vitro co‐cultured with CFSE‐stained spleen cells (Figure 4E). After 3 days of coculture, we observed that WT BMDCs induced greater proliferation of T cells and B cells than *Trem2* ^−/−^ BMDCs (Figure 4E). Furthermore, we detected the expression of activation markers on T cells (CD25 and CD69) and B cells (MHC II and CD69) following coculture, which showed significantly higher levels after co‐culture with WT BMDCs compared to *Trem2* ^−/−^ BMDCs (Figure 4F). In addition, we verified the observation in DCs isolated from the peripheral blood of SLE patients. These DCs were pretreated with either anti‐hTREM2 blocking antibody (anti‐hTREM2) or control IgG, followed by NETs stimulation. As expected, anti‐hTREM2 treatment reduced the expression of MHC II and CD86 on DCs (Figure S10A, Supporting Information). And then, these pretreated DCs were cocultured with PBMCs for 3 days. Flow cytometry analysis revealed that, upon NETs stimulation, DCs blocked with anti‐hTREM2 induced significantly lower proliferation and activation levels in both T and B cells compared to those treated with the IgG control (Figure S10B–D, Supporting Information). Taken together, these data indicated that TREM2 drives NETs‐induced DCs maturation and antigen presentation via promoting NETs phagocytosis.

### 2.5 TREM2 Interacts with MPO to Promote DCs Maturation and Phagocytosis

**Figure 5:**
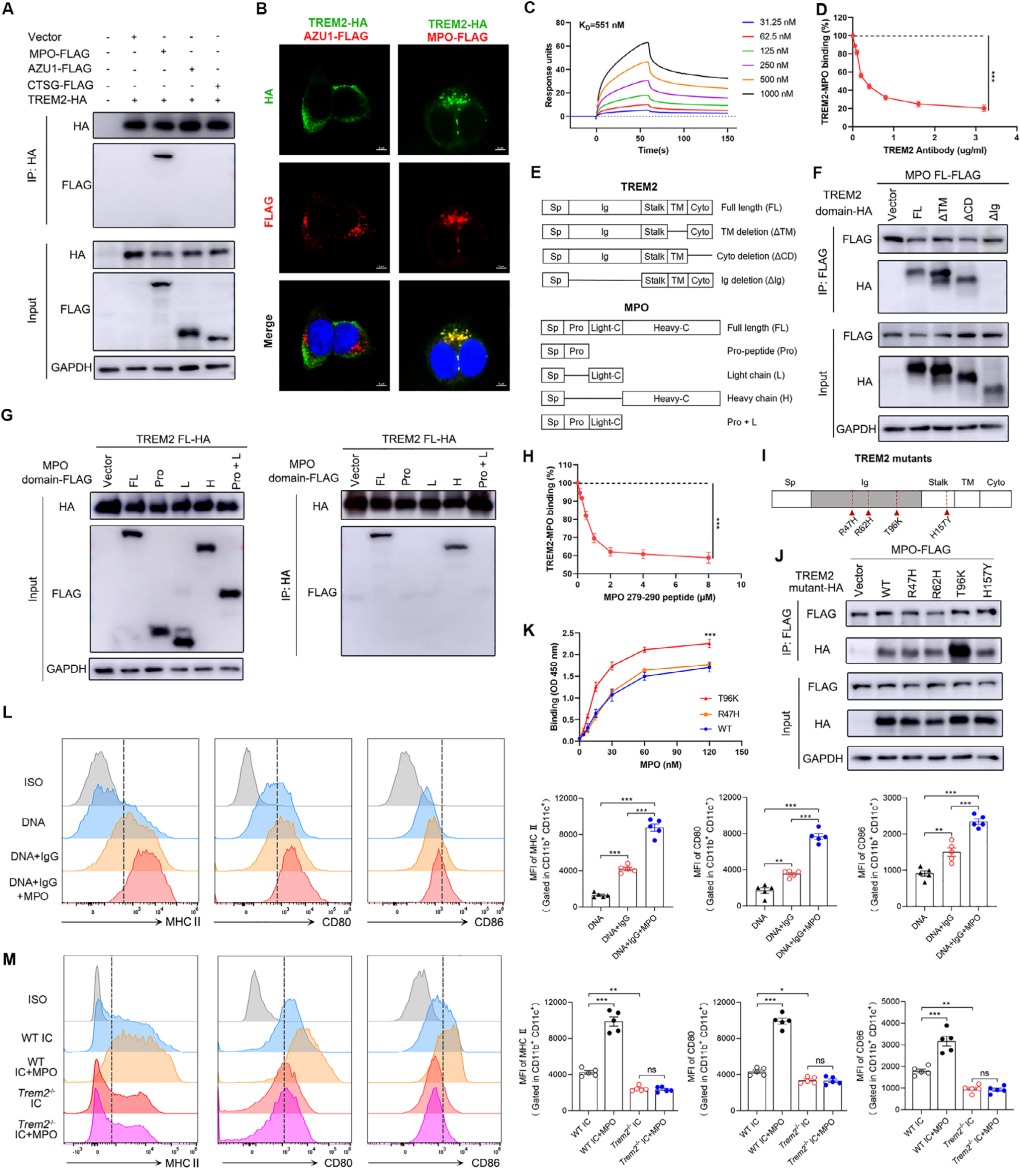
TREM2 interacts with MPO to promote DCs maturation. A) 293T cells were transfected with PcDNA3.1 vector containing HA‐tagged TREM2 and FLAG‐tagged MPO, or FLAG‐tagged AZU1, or FLAG‐tagged CTSG. Blots of cell lysates (Input) or anti‐HA IP were analyzed by Western blotting for HA, FLAG. B) 293T cells were transfected with a PcDNA3.1 vector containing HA‐tagged TREM2 and FLAG‐tagged MPO or AZU1. Immunofluorescence staining of HA, FLAG and DAPI in 293T cells to analyze colocalization of TREM2 and MPO or AZU1. Scale bar, 5 µm. C) The binding profile of TREM2 to different concentrations of MPO was measured by surface plasmon resonance (SPR) assay. D) Blocking binding experiments were performed to measure the binding of TREM2 to MPO in the presence of TREM2 antibody (n=5, one‐way ANOVA). E–J) The PcDNA3.1 plasmid encoding HA‐tagged WT (full length, FL), or truncated forms of TREM2, respectively, deleting the transmembrane (ΔTM), the cytosolic domain (ΔCD), or Ig (ΔIg), as well as 4 mutant forms of TREM2 (R47H, R62H, T96K, and H157Y) were constructed. The PcDNA3.1 plasmid encoding FLAG‐tagged FL, or pro‐peptide (Pro), or light chain L), or heavy chain H) of MPO was constructed. (F,G) 293T cells were transfected with FL or truncated forms of TREM2 plasmids and FL or the critical domain of MPO plasmids. Co‐IP assay was performed to identify the key domains mediating the interaction between TREM2 and MPO. H) Competitive binding experiment was performed to measure the binding of TREM2 to MPO in the presence of peptide encompassing residues 279‐290 of MPO (n=5, one‐way ANOVA). I,J) 293T cells were transfected with WT or mutant TREM2 plasmids and MPO domain plasmids. The Co‐IP assay was performed to analyze the interaction between mutant TREM2 and MPO. K) Binding of MPO to immobilized WT, R47H, T96K TREM2 was determined by solid‐phase binding (SPB) assay (n=5, one‐way ANOVA). (L) WT BMDCs were stimulated with DNA, or ICs (DNA+IgG), or ICs+MPO for 24 h. The maturation markers (MHC II, CD80, and CD86) of BMDCs were analyzed by flow cytometry (n=5, one‐way ANOVA). (M) WT or *Trem2* ^−/−^ BMDCs were stimulated with ICs (DNA+IgG), or ICs+MPO for 24 h. The maturation markers of BMDCs from four groups were analyzed by flow cytometry (n=5, one‐way ANOVA). The results are presented as the mean ± SEM from at least three independent experiments. ^*^ *P* < 0.05; ^**^ *P* < 0.01; ^***^ *P* < 0.001; ns, no significance difference.

NETs are a complex composed of DNA, histones, and granule protein.^[^ ^31^ ^]^ To explore the specific component of NETs binding to TREM2, we applied mass spectrometry (MS) and Co‐immunoprecipitation (Co‐IP) to identify the potential ligand for TREM2. The MS results identified MPO, azurocidin (AZU1), and cathepsin G (CTSG) in NETs as the top three non‐histones interacting with TREM2 (Table S2, Supporting Information). Moreover, we transfected 293T cells with HA‐tagged TREM2 and FLAG‐tagged MPO, or FLAG‐tagged AZU1, or FLAG‐tagged CTSG plasmids, and then assessed for protein interaction. Co‐IP data revealed that TREM2 interacted with the MPO, but not AZU1 or CTSG (**Figure** **5**A). Immunofluorescence staining also exhibited that TREM2 and MPO could colocalization in 293T cells transfected with HA‐tagged TREM2 and FLAG‐tagged MPO plasmids (Figure 5B). The surface plasmon resonance (SPR) assay showed a dose‐dependent binding of MPO to the TREM2 recombinant protein (Figure 5C). Furthermore, the specificity of this interaction was confirmed by blocking experiments using an anti‐TREM2 antibody, which effectively inhibited the binding (Figure 5D).

As a transmembrane receptor, TREM2 contains an extracellular Ig‐like domain, a transmembrane domain, and a short cytoplasmic tail.^[^ ^32^ ^]^ To explore the critical domains of TREM2 that interacted with MPO, we constructed plasmids encoding full‐length (FL) TREM2, truncated forms of TREM2 lacking the transmembrane (ΔTM), the cytosolic domain (ΔCD), and the extracellular Ig domain (ΔIg), respectively (Figure 5E). Results showed that the interaction between TREM2 and MPO disappeared when the Ig domain was deleted (Figure 5F), suggesting that TREM2 bound to MPO through the extracellular Ig domain. In addition, we investigated the specific domain of MPO involved in the interaction with TREM2. MPO mainly consists of pro‐peptide (Pro), light chain (L), and heavy chain (H).^[^ ^33^ ^]^ We constructed plasmids encoding each of these domains (Figure 5E) and evaluated their binding capacity with TREM2. Co‐IP assay data demonstrated that the heavy chain of MPO was able to interact with TREM2, but the pro‐peptide and light chain failed (Figure 5G). The molecular docking analysis also revealed a potential binding interface between the Ig domain of TREM2 (19–118 aa) and the heavy chain of MPO (279–745aa), and the residues encompassing 279–290 of the MPO peptide chain were predicted to mediate the binding of MPO to the TREM2 through hydrogen bonding (Figure S11, Supporting Information). The competitive binding assay confirmed that a peptide containing MPO residues 279–290 significantly blocked full‐length MPO binding to TREM2, indicating that the 279–290 region of MPO is required for MPO‐TREM2 interaction (Figure 5H). Previous studies reported that TREM2 variants alter the binding of TREM2 to its ligands ^[^ ^34^ ^]^. Thus, we constructed TREM2 plasmids with the mutations of various conserved residues, including R47H, R62H, T96K, and H157Y (Figure 5I), which are crucial for ligand binding and signal transduction.^[^ ^34^ ^]^ The results showed that the T96K mutation enhanced the binding between TREM2 and MPO (Figure 5J). Similarly, the solid‐phase binding assay showed that the T96K mutation bound to MPO with a 1.3 fold higher affinity than the WT or R47H TREM2 protein (Figure 5K). Consistent with this, structural analysis also indicated that the T96K mutation significantly increased the binding ability of TREM2 to MPO (Figures S12 and S13, Supporting Information).

After identifying the NETs‐derived MPO as a novel ligand of TREM2 in structure, we then explored its function. Previous study demonstrated that NETs containing neutrophil proteins such as LL37 and HNP are required for the ability of ICs to activate immune cells in SLE ^[^ ^35^ ^]^. Thus, we first investigated the role of MPO in facilitating the ICs to activate DCs. The results showed that BMDCs with ICs stimulation expressed higher levels of activation markers (MHC II, CD80, and CD86) compared to free DNA stimulation (Figure 5L). When MPO protein was added to ICs, the complexes further increased the levels of activation markers in BMDCs (Figure 5L), suggesting that MPO promoted the ICs to activate DCs. Next, we explored whether the function of MPO was mediated by TREM2. The *Trem2* deficiency in BMDCs abolished the increase in levels of activation markers after stimulation with the complexes of ICs and MPO (Figure 5M). Similarly, we found that MPO could facilitate the uptake of DNA‐containing ICs by BMDCs (Figure S14A, Supporting Information). However, the effect of MPO was abrogated in *Trem2*‐deficient BMDCs (Figure S14B, Supporting Information). Taken together, these data indicated that TREM2 interacted with MPO and promoted ICs to enter and activate DCs.

### 2.6 NETs‐Induced DAP12/SYK and cGAS/STING Signaling Activation is Dependent on TREM2 Receptor

**Figure 6:**
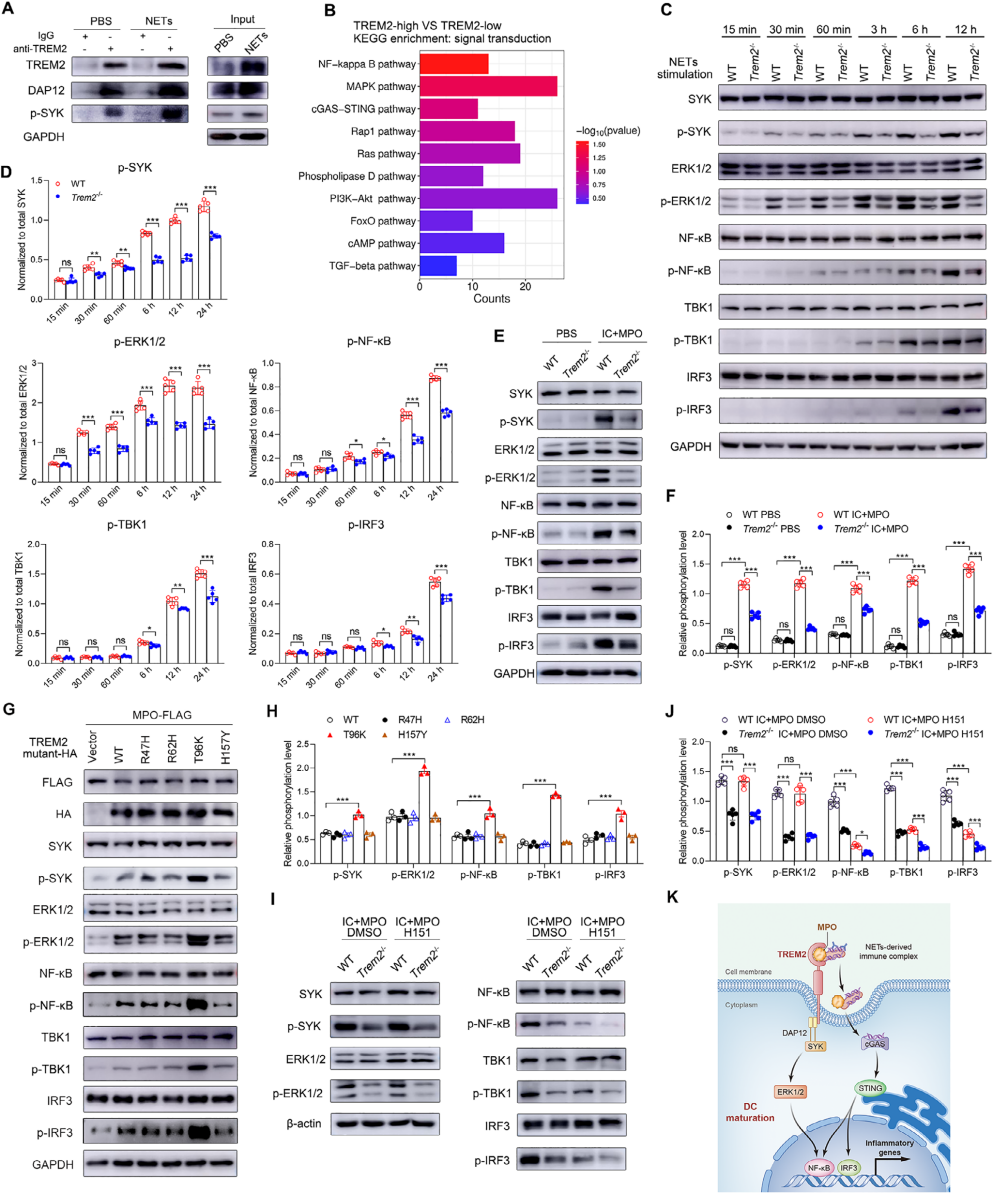
NETs activate DAP12/SYK and cGAS/STING signaling pathway via TREM2. A) BMDCs were stimulated with PBS or NETs for 12h and then were lysed. Anti‐TREM2 IP were analyzed for DAP12 and p‐SYK. B) KEGG pathway enrichment analysis was performed on the differentially expressed genes relating to signal transduction that were identified between SLE patients with high and low TREM2 expression. C) WT and *Trem2* ^−/−^ BMDCs were stimulated with NETs at multiple time points (15 min, 30 min, 60 min, 3 h, 6 h, and 12 h). The protein levels of total SYK, ERK1/2, NF‐κB, TBK1, IRF3, along with their phosphorylated forms were determined by WB. D) The band intensities of p‐SYK, p‐ERK1/2, p‐NF‐κB, p‐TBK1, and p‐IRF3 were quantified using ImageJ software and normalized to their corresponding total protein levels (n=5, unpaired 2‐tailed Student's *t*‐test). E,F) WT and *Trem2* ^−/−^ BMDCs were stimulated with PBS or IC+MPO for 12 h. The protein levels of total and phosphorylated SYK, ERK1/2, NF‐κB, TBK1, and IRF3 were determined by WB. The band intensities were quantified and normalized to their corresponding total protein levels (n=5, one‐way ANOVA). G,H) Human DCs were transfected with HA‐tagged mutant TREM2 plasmids (WT, R47H, R62H, T96K, H157Y) and FLAG‐tagged MPO plasmid, and then stimulated with ICs for 12 h. The protein levels of total and phosphorylated SYK, ERK1/2, NF‐κB, TBK1, and IRF3 were determined by WB. The band intensities were quantified and normalized to their corresponding total protein levels (n=3, One‐way ANOVA). I,J) WT and *Trem2* ^−/−^ BMDCs were pretreated with STING inhibitor (H151) or DMSO, and then the four groups were stimulated with IC+MPO for 12 h. The protein levels of total and phosphorylated SYK, ERK1/2, NF‐κB, TBK1, and IRF3 were determined by WB. The band intensities were quantified and normalized to their corresponding total protein levels (n=5, One‐way ANOVA). K) The schematic diagram of TREM2‐mediated downstream signaling induced by NETs/MPO. The results are presented as the mean ± SEM from at least three independent experiments. ^*^ *P* < 0.05; ^**^ *P* < 0.01; ^***^ *P* < 0.001; ns, no significance difference.

The above data indicate that NETs‐derived MPO is a new ligand for TREM2. Next, we applied Co‐IP and western blot to explore the downstream signaling molecules of TREM2 after NETs/MPO stimulation. It has been reported that upon ligand binding, TREM2 recruits SYK via DAP12, leading to SYK and ERK1/2 phosphorylation, thereby promoting DC maturation and phagocytosis.^[^ ^20^, ^21^ ^]^ To investigate whether TREM2 regulates a similar signaling pathway in response to NETs stimulation, anti‐TREM2 IP was performed. NETs treatment enhanced the interaction between TREM2 and DAP12/p‐SYK in BMDCs compared to the PBS‐treated group (**Figure** **6**A). The immunoblot analysis showed that *Trem2* ^−/−^ BMDC with NETs stimulation exhibited lower levels of p‐SYK and p‐ERK1/2 compared to the WT group with NETs stimulation (Figure S15A,B, Supporting Information), suggesting that TREM2 mediates DAP12/SYK/ERK1/2 signaling activation. Furthermore, the KEGG pathway analysis revealed that NF‐κB, MAPK, and cGAS/STING pathways were the top three enriched pathways in SLE patients with high TREM2 expression compared to those with low expression (Figure 6B), indicating that other pathways also participate in the downstream signaling of TREM2.

Previous studies reported that NETs activate the intracellular cGAS/STING pathway after being endocytosed.^[^ ^36^ ^]^ cGAS is a crucial intracellular PRR that functions to detect cytosolic DNA.^[^ ^37^ ^]^ First, we detected the expression levels of cytoplasmic PRRs involved in lupus immunopathology.^[^ ^38^ ^]^ The results showed that mRNA levels of cGAS/STING were significantly lower in *Trem2* ^−/−^ BMDCs than in WT BMDCs (Figure S15C, Supporting Information), indicating that *Trem2* deficiency impairs the cGAS/STING signaling pathway. Next, we assessed the relationship between DAP12/SYK and cGAS/STING. We analyzed the activation kinetics of these two pathways at multiple time points (15, 30, and 60 min, 3, 6, and 12 h) in NETs‐stimulated WT and *Trem2* ^−/−^ BMDC. The results showed that p‐SYK and p‐ERK1/2 were detected at early time points (15–60 min). Compared to WT BMDC, *Trem2* deficiency reduced the activation levels of p‐SYK and p‐ERK1/2 at 60 min, respectively. Analysis of the cGAS/STING pathway showed that its key components, p‐TBK1 and p‐IRF3, were detected at ≈3 h post‐stimulation. Similarly, *Trem2* deficiency markedly reduced the levels of p‐TBK1 and p‐IRF3 at 12 h, indicating impaired activation of TBK1 and IRF3 (Figure 6C, D). Taken together, these results revealed a sequential activation. TREM2‐dependent SYK/ERK1/2 signaling axis was rapidly activated at early time points upon NETs treatment, followed by activation of the intracellular cGAS/STING/TBK1/IRF3 pathway at ≈3 h post‐stimulation. Notably, NF‐κB activation also appeared. The phosphorylated NF‐κB was initially detected at early time points, and its signal was subsequently enhanced after TBK1 activation (Figure 6C, D), indicating that it is synergistically regulated by both pathways.

As NETs‐derived MPO is a ligand that directly binds to TREM2, we also explored its function in regulating TREM2‐mediated signaling. Following stimulation with ICs+MPO, *Trem2*‐deficient BMDCs showed attenuated activation of key molecules in both the DAP12/SYK and cGAS/STING signaling pathways, including p‐SYK, p‐ERK1/2, p‐NF‐κB, p‐TBK1, and p‐IRF3, compared to WT BMDCs (Figure 6E, F). Since our data showed that the T96K mutation in TREM2 enhanced its binding to MPO (Figure 5I–K), we further investigated whether the mutations influenced the activation of these signaling pathways. Human DCs were transfected with plasmids encoding HA‐tagged TREM2 (WT, or R47H, R62H, T96K, H157Y) and FLAG‐tagged MPO, and then stimulated with ICs. Consistent with the enhanced interaction between the TREM2 mutation (T96K) and MPO (Figure 5I–K), the phosphorylation levels of SYK, ERK1/2, NF‐κB, TBK1, and IRF3 were also increased in DCs transfected with a plasmid encoding the T96K TREM2 mutation compared to those transfected with a WT TREM2 plasmid (Figure 6G, H). Furthermore, to verify the causal relationship between DAP12/SYK and cGAS/STING signaling pathways, we used the specific STING inhibitor H151. Inhibition of STING significantly blocked downstream TBK1 and IRF3 activation but had no effect on the SYK and ERK1/2 activation (Figure 6I, J), indicating that the activation of cGAS/STING is later than the classical DAP12/SYK signaling. Overall, these data demonstrated that TREM2 mediates NETs/MPO‐induced DAP12/SYK and cGAS/STING signaling activation (Figure 6K). In sequence, TREM2/DAP12/SYK signaling is an early event triggered by external NETs binding, which promotes phagocytosis and subsequent intracellular cGAS/STING pathway activation.

### 2.7 Inhibition of NETs or MPO Protects Against TREM2‐Driven Lupus Progression

**Figure 7:**
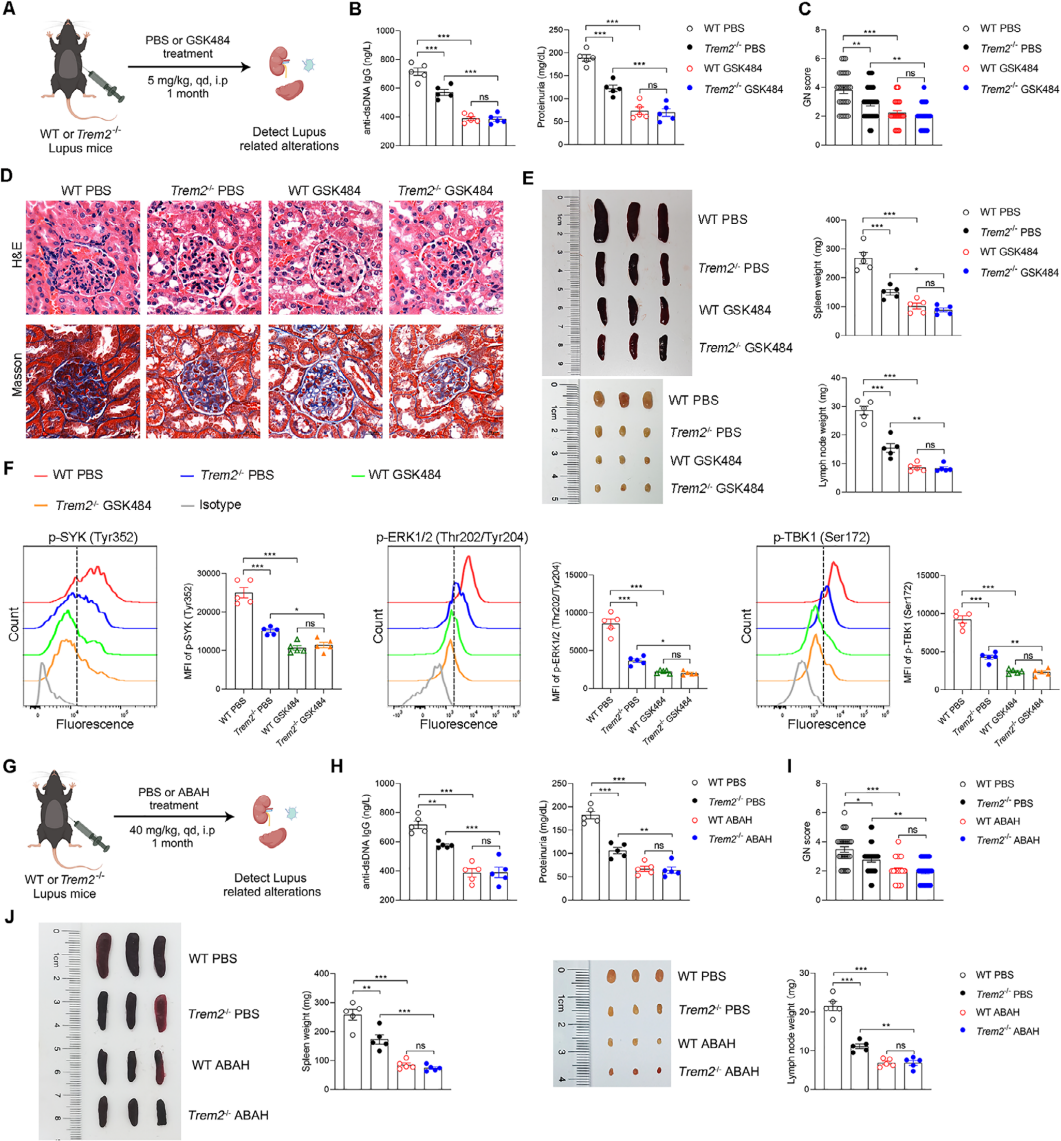
Inhibition of NETs and MPO protects against TREM2‐driven lupus progression. A) WT or *Trem2* ^−/−^ lupus mice were administered with NETs inhibitor (GSK484) or PBS by intraperitoneal injection (i.p.) every day for one month. B) The serum and urine samples from each mouse of the indicated groups were collected after DNase I or PBS treatment. The anti‐dsDNA Abs and proteinuria levels were detected by ELISA (n=5, one‐way ANOVA). C,D) The paraffin‐embedded kidney sections were stained with H&E and Masson. The Kidney pathology was evaluated by GN scores (n=30, one‐way ANOVA). Scale bar, 20 µm. E) The gross appearance and the weight of spleens or lymph nodes from mice of the indicated groups were recorded (n=5, one‐way ANOVA). F) The phosphorylation levels of SYK (Tyr352), ERK1/2 (Thr202/Tyr204), and TBK1(Ser172) in splenic DCs were quantified by flow cytometry (n=5, one‐way ANOVA). G) WT or *Trem2* ^−/−^ lupus mice were administered with MPO inhibitor (ABAH) or PBS by i.p. every day for one month. H) The anti‐dsDNA Abs and proteinuria levels were detected by ELISA (n=5, one‐way ANOVA). I) The Kidney pathology was evaluated by GN scores (n=30, one‐way ANOVA). J) The gross appearance and the weight of spleens or lymph nodes were recorded (n=5, one‐way ANOVA). The results are presented as the mean ± SEM from three separate experiments. ^*^ *P* < 0.05; ^**^ *P* < 0.01; ^***^ *P* < 0.001; ns, no significance difference.

NETs play a critical role as autoantigens in the immunopathology of SLE, and evidence suggests that NETosis is significantly upregulated, leading to the accumulation of substantial amounts of NETs in SLE patients.^[^ ^8^ ^]^ To further explore whether NETs are required for TREM2‐driven lupus pathogenesis, we treated both WT and *Trem2* ^−/−^ lupus mice with GSK484, a PAD4 inhibitor that blocks NET formation (**Figure** **7**A). Upon GSK484 treatment, both mouse strains exhibited decreased levels of serum anti‐dsDNA Abs and proteinuria compared to their respective PBS‐treated control group (Figure 7B). Moreover, those markers showed no significant differences between the GSK484‐treated WT and *Trem2* ^−/−^ lupus mice (Figure 7B). Similarly, administration of GSK484 also led to a marked amelioration of lupus nephritis pathology in both mouse strains (Figure 7C, D). GSK484 treatment significantly reduced the size and weight of the spleen and lymph nodes in both WT and *Trem2* ^−/−^ lupus mice compared to their respective PBS‐treated controls, and there was no significant difference between the two groups with GSK484 treatment (Figure 7E). To investigate the underlying mechanism, we assessed the activity of the TREM2 signaling pathway. Flow cytometric analysis revealed that GSK484 suppressed the phosphorylation of SYK, ERK1/2, and TBK1 compared to the PBS control (Figure 7F), indicating that NET inhibition blocks TREM2‐mediated DAP12/SYK and cGAS/STING signaling activation. These data demonstrate that NETs are essential for TREM2‐driven lupus manifestations and signaling activation. As SLE patients have an impaired ability to degrade NETs,^[^ ^3^ ^]^ the accumulated NETs‐derived DNA acts as an autoantigen that is internalized via TREM2‐mediated phagocytosis. To further validate the role of NETs, we also administered DNase I (degradation agent of DNA) to WT and *Trem2* ^−/−^ lupus mice (Figure S16A, Supporting Information). Administration of DNase I significantly ameliorated key disease manifestations in both WT and *Trem2* ^−/−^ lupus mice, including serum anti‐dsDNA Abs, proteinuria, lupus nephritis pathology, and lymphoid organ enlargement, compared to PBS‐treated controls (Figure S16B–D, Supporting Information). Notably, the therapeutic effect of DNase I did not differ significantly between the two genotypes (Figure S16B–D, Supporting Information). Taken together, these results from two experimental approaches demonstrate that NETs are essential for TREM2‐driven lupus pathogenesis.

Having identified NETs‐derived MPO as a TREM2 ligand, we further assessed its functional role in TREM2‐driven lupus progression by administering the MPO inhibitor 4‐Aminobenzohydrazide (ABAH) to WT and *Trem2* ^−/−^ lupus mice (Figure 7G). ABAH treatment significantly rescued lupus‐related manifestations—including proteinuria, serum anti‐dsDNA Abs levels, kidney pathology, splenomegaly, and lymphadenopathy—in both genotypes compared to PBS‐treated controls (Figure 7H–J). Crucially, the therapeutic efficacy of ABAH was comparable between WT and *Trem2* ^−/−^ lupus mice (Figure 7H–J). Collectively, these results demonstrated that NETs‐derived MPO is required for TREM2‐driven lupus progression.

## 3 Discussion

SLE is an autoimmune disease characterized by hyperactive immune cells, aberrant production of proinflammatory cytokines and autoantibodies, and consequent inflammatory organ damage.^[^ ^3^ ^]^ While B‐cell overactivation and autoantibody production are central to lupus immunopathogenesis ^[^ ^39^ ^],^ the partial efficacy of the B‐cell‐regulating drug Belimumab^[^ ^40^ ^]^ implies contributions from other immune mechanisms. A growing body of evidence indicates that DCs are essential in autoimmune responses, and DC dysfunction contributes to autoimmune diseases, including SLE and autoimmune encephalomyelitis.^[^ ^41^, ^42^ ^]^ In SLE patients, DCs exhibit abnormal activation and maturation after being exposed to autoantigen.^[^ ^43^ ^]^ Activated DCs present autoantigens to T cells and directly promote B cell survival through cytokine secretion, thereby further amplifying the inflammatory response.^[^ ^11^ ^]^ Notably, conventional DCs (cDCs), including cDC1 and cDC2 subsets, play distinct roles in SLE.^[^ ^41^ ^]^ cDC1s are particularly efficient at cross‐presenting antigens and activating CD8⁺ T cells. Meanwhile, cDC2s are specialized in presenting autoantigens via MHC II to prime CD4⁺ T cells and drive T helper cell responses, thereby further promoting B cell activation and autoantibody production.^[^ ^44^ ^]^ Therefore, therapeutic strategies targeting DC function represent a promising avenue for SLE treatment.

Previous in vitro studies indicated that TREM2 promotes DC maturation and activation.^[^ ^21^, ^45^ ^]^ Another in vivo study showed that TREM2 enhances the function of DCs in triggering an inflammatory response and then exacerbates colonic inflammation.^[^ ^22^ ^]^ In this study, we identified that TREM2 expression is elevated on DCs from SLE patients, particularly within the cDC2 subset. Using the murine lupus model, BMDCs and patient samples, we confirmed that TREM2 potentiates DC maturation and activation. Furthermore, we investigated its impact on antigen presentation and found that TREM2 enhances the capacity of DCs to drive CD4⁺ T cell proliferation. Consistent with its expression profile, TREM2 signaling favored the differentiation of T cells into Th2 and Th17 subsets and promoted B cell activation. Consequently, we demonstrated that TREM2 aggravates lupus‐like symptoms in mice by enhancing DC maturation and antigen presentation.

TREM2, a transmembrane receptor, can undergo alternative splicing or proteolytic processing to produce sTREM2, which serves as a predictive factor for the development of Alzheimer's disease (AD) and dementia.^[^ ^46^ ^]^ Moreover, it has been shown to possess biological activity, such as binding to amyloid aggregates and exerting a beneficial effect on Aβ pathology^[^ ^47^ ^]^. Evidence from autoimmune diseases of the central nervous system also suggests a potential functional role. For instance, elevated cerebrospinal fluid (CSF) sTREM2 levels have been reported in multiple sclerosis^[^ ^48^ ^]^ and are associated with microglial dysfunction in neuromyelitis optica spectrum disorders.^[^ ^49^ ^]^ Notably, in neuropsychiatric SLE, increased CSF sTREM2 levels were observed in patients with an acute confusional state.^[^ ^50^ ^]^ In the present study, we revealed that serum sTREM2 levels were increased in SLE patients and were positively correlated with clinical markers of disease activity and severity. However, based solely on our correlative data, we cannot conclude whether sTREM2 is merely a surrogate biomarker reflecting systemic immune activation or if it plays an active pathogenic or protective role in SLE. Therefore, future mechanistic studies are essential to determine its specific biological functions and its role in SLE pathogenesis.

TREM2 is a pivotal modulator of innate immunity, with its diverse roles influencing the progression of multiple diseases. In the nervous system, it critically regulates microglial functions—such as proliferation, activation, and phagocytosis—promoting a protective phenotype essential for clearing cellular debris and amyloid‐beta plaques and maintaining neural homeostasis.^[^ ^34^, ^51^, ^52^ ^]^ Similarly, in macrophages, TREM2 enhances phagocytosis by recognizing lipids, apoptotic cells, and pathogens, thereby playing a role in atherosclerosis and infectious diseases.^[^ ^53^, ^54^ ^]^ In autoimmune disease, TREM2 has been reported to drive the development of multiple sclerosis and arthritis.^[^ ^55^, ^56^ ^]^ Regarding its role in DCs, in vitro and in vivo studies demonstrated that TREM2 promotes DC maturation and survival,^[^ ^21^, ^22^ ^]^ a role our current study confirms in the context of lupus progression. However, the role of TREM2 in regulating immune cell inflammation remains controversial. For instance, it negatively regulates DC maturation in liver ischemia‐reperfusion injury^[^ ^23^ ^]^ and suppresses macrophage responses during mycobacterial infection to promote bacterial evasion.^[^ ^57^ ^]^ The observations suggested that the TREM2 functions depend on the specific cell type, ligands, and the pathogenesis of diseases.

As a surface receptor, TREM2 exerts multiple functions by binding to a range of ligands, including bacterial products, phospholipids, lipoproteins, and proteins ^[^ ^20^ ^],^ but the highly specific TREM2 ligand is largely unknown in the immunopathogenesis of SLE. Given that autoantigens are the triggering signals of autoimmunity, we aimed to identify specific autoantigens relevant to TREM2 signaling in SLE. Our results demonstrated that NETs acted as key autoantigens associated with TREM2. Mechanistically, using a combination of murine models and human samples, we demonstrated that TREM2 mediates NETs‐induced DC maturation and antigen presentation by facilitating the phagocytosis of NETs. Accumulating evidence supports the implication of NETs in the pathogenesis of SLE.^[^ ^35^, ^58^ ^]^ The prevailing model suggests that NETs‐derived nucleic acids activate intracellular sensors. NETs can activate pDCs to produce type I interferons in a DNA‐ and TLR9‐dependent manner,^[^ ^9^ ^]^ while NETs‐associated RNA can trigger TLR7 signaling in endothelial cells.^[^ ^59^ ^]^ Furthermore, oxidized mitochondrial DNA from NETs may engage the cytosolic DNA sensor cGAS.^[^ ^60^ ^]^ Observations from other autoimmune contexts, such as ANCA‐associated vasculitis, also indicate that NETs can activate myeloid DCs ^[^ ^61^ ^],^ yet the specific receptor mediating this effect remained unknown. Here, our study uncovers a previously unrecognized pathway centered on TREM2, which directly binds to NETs and facilitates their phagocytosis by DCs. This process is distinct from TLR‐driven innate interferon responses and instead directly bridges NETs recognition to the activation of adaptive immunity through DC antigen presentation. Thus, our discovery not only provides a mechanistic explanation for earlier observations of NETs‐mediated DC activation but also positions the TREM2 pathway as a key contributor to SLE pathogenesis.

NETs are complexes composed of nucleic acids and proteins.^[^ ^7^ ^]^ To investigate the specific component of NETs binding to TREM2, we applied MS analysis, Co‐IP, and immunofluorescence assays to demonstrate that MPO can directly interact with TREM2. A previous study reported that the T96K TREM2 variant increases the binding to phospholipids.^[^ ^62^ ^]^ Furthermore, we found that the T96K TREM2 variant enhanced the interaction with MPO. Taken together, these data elucidate that MPO is a new ligand for TREM2 in SLE. MPO is an important peroxidase primarily expressed in neutrophils, which is involved in multiple inflammatory diseases by inducing oxidative stress and activating immune cells.^[^ ^63^, ^64^, ^65^ ^]^ In SLE patients, MPO levels positively correlate with disease severity.^[^ ^66^ ^]^ However, the function of the binding between MPO and TREM2 remains unclear. Several studies reported that the neutrophil proteins (LL37 and HNP) are required for the ability of DNA contained in NETs to activate pDCs. These proteins promote the uptake and recognition of DNA by pDCs.^[^ ^9^, ^35^, ^67^ ^]^ Herein, we revealed that MPO facilitates the DNA to enter and activate DCs by directly binding to TREM2. In addition, studies have reported that MPO promotes NETs formation, and MPO inhibition reduces both NETs formation and kidney damage in patients with ANCA‐associated vasculitis.^[^ ^68^ ^]^ To verify the role of NETs and MPO in TREM2‐aggravated lupus progression, *Trem2* ^−/−^ and WT lupus mice were administered with GSK484 (inhibition the NETs formation) or ABAH (MPO inhibitor). The GSK484 or ABAH totally abrogated the differences in lupus manifestations between the two genotype lupus mice, implying that NETs and MPO are required for TREM2‐mediated lupus. Collectively, we reveal the function of NETs‐derived MPO as a ligand for TREM2, and provide a new therapeutic target for lupus.

TREM2 is known to transmit signals through the cytoplasmic adapter protein DAP12, which is primarily expressed on DCs and macrophages.^[^ ^20^ ^]^ DAP12 mediates the functions of TREM2, such as phagocytosis and microglial activation, by recruiting SYK tyrosine kinases and activating downstream signaling pathways such as ERK and PI3K.^[^ ^69^, ^70^ ^]^ In our study, we demonstrated that TREM2‐mediated DC maturation and phagocytosis of NETs depend on the DAP12/SYK/ERK1/2 signaling pathway. Following the engulfment of NETs by DCs, the cytosolic DNA sensor cGAS recognizes NETs‐derived DNA. This recognition activates the STING signaling pathway, which in turn activates TBK1, IRF3, and NF‐κB. Previous studies also reported that NETs can be taken up by various cells through endocytosis and then activate the cGAS/STING pathway.^[^ ^36^, ^37^, ^71^ ^]^ However, what's important and distinctive is that our study emphasizes the role of membrane receptor TREM2 in NETs‐induced cGAS‐STING pathway, in which TREM2 enhances the uptake of NETs by DCs. Taking these data together, we demonstrated that TREM2 mediates the sequential activation of two signaling pathways in response to NETs. First, it directly activates TREM2‐mediated DAP12/SYK/ERK1/2 signaling. Subsequently, TREM2 facilitates the engulfment of NETs, leading to activation of the cGAS/STING signaling pathway in DCs.

In terms of the regulatory role of TREM2 in disease, we employed multiple murine lupus models—including ALD‐DNA‐induced, pristane‐induced, and DC transfusion models—to demonstrate that TREM2 exacerbated lupus progression by promoting DC maturation. This finding was further validated by administering a TREM2 inhibitory antibody in spontaneous MRL/lpr lupus mice. To further validate the role of TREM2 in a human‐relevant context, we utilized the humanized anti‐TREM2 antibody PY314.^[^ ^72^ ^]^ Experiments on DC from SLE patients revealed that TREM2 blockade significantly impaired their maturation and antigen‐presenting capacity in response to NETs. These findings from patient‐derived samples not only confirm the pathophysiological role of TREM2 in human SLE but also highlight the translational value of TREM2 blockade, as it effectively modulates DC‐driven adaptive immune responses in a human disease context. In summary, our study provides mechanistic insights into lupus pathogenesis and proposes a promising therapeutic strategy combining TREM2 blockade with NET elimination.

## 4 Experimental Section

### Human Participants and PBMCs Isolation

SLE patients (n═46) and age‐matched healthy controls (n═30) were recruited from The Fifth Affiliated Hospital of Sun Yat‐sen University (Zhuhai, China). All participants provided written informed consent prior to enrollment in the study. The consent process was conducted by the attending physicians, who explained the purpose of the research, the procedures involved, potential risks and benefits, and the confidentiality of their personal data. Participants were informed that their participation was entirely voluntary and that they could withdraw at any time without affecting their medical care. The study was conducted in accordance with the Declaration of Helsinki and under protocols approved by the Ethics Committee of The Fifth Affiliated Hospital of Sun Yat‐Sen University (approval number: 2024‐K93‐1). Disease activity was assessed by the SLEDAI‐2K on the day of blood collection. The diagnosis of SLE was based on the 2019 EULAR/ACR classification criteria. The exclusion criteria included SLE patients complicated with recent or active infection, malignancy, and other autoimmune diseases. Clinical and laboratory characteristics of SLE patients and healthy controls are provided in Table S1 (Supporting Information). Blood from SLE patients and healthy controls was collected using lithium heparin tubes, centrifuged at 3000 rpm for 10 min, and plasma aliquots were stored at −80 °C. PBMCs were isolated by a lymphocyte separation solution (TBD Sciences).

### Human Neutrophils Stimulation and Isolation of NETs

Heparinized blood from SLE patients and healthy controls was centrifuged by Polymorphprep (Axis‐Shield) for 35 min at 500 g to obtain neutrophils. Neutrophils from two groups were respectively seeded on coverslips coated with Poly‐L‐lysine, and cultured in complete RPMI 1640 medium supplemented with 2% autologous serum at a density of 2 × 10^6^ cells mL^−1^. Anti‐RNP IgG (10 µg mL^−1^) purified from SLE patients with a high titer of anti‐RNP antibodies was added to the culture, and neutrophils were stimulated for 4 h. In some experiments, the stimulated cells were fixed and permeabilized for immunofluorescence staining. The cells were stained with the following antibodies: rabbit anti‐human citrullinated histone H3 (citH3) antibody (Abcam), mouse anti‐human MPO (Abcam), followed by AF594‐labeled goat anti‐rabbit IgG (Invitrogen), AF488‐labeled goat anti‐mouse IgG (Invitrogen), and DAPI (Beyotime). Images were captured with a Zeiss 880 confocal microscope.

To isolate NETs from the activated neutrophils, the cell culture medium was gently aspirated and discarded so that the NETs and neutrophil layers stuck to the bottom. Cells were washed twice with ice‐cold PBS to remove all adhesions from the bottom of the petri dish. The PBS containing NETs and neutrophils was centrifuged at 450 g for 10 min at 4 °C to obtain cell‐free supernatants. Then the supernatant was centrifuged at 18 000 g for 15 min at 4 °C. The supernatant was discarded, and the remaining product was resuspended with ice‐cold PBS. The DNA concentration of supernatants containing NETs was measured using a NanoDrop (Thermo Fisher Scientific). Use immediately or store frozen at −80 °C.

### Mice

WT C57BL/6 mice were purchased from the Laboratory Animal Center of Guangdong Province. CD45.1 C57BL/6 mice were procured from Nanjing GemPharmatech. The lupus‐prone MRL/Lpr mice were purchased from Changzhou Cavens Model Animal Center. *Trem2* ^−/−^ C57BL/6 mice were provided by Marco Colonna (Washington University School of Medicine). The mice with loxP‐flanked alleles of *Trem2* exon 2/3 (*Trem2* ^fl/fl^) were generated in the Model Animal Research Center (MARC) of Nanjing University. Mice were backcrossed to the C57BL/6J background for more than 6 generations. To generate mice with a CD11c‐specific knockout of the *Trem2* alleles (*Trem2* ^fl/fl^ CD11c‐Cre), *Trem2* ^fl/fl^ mice were crossed with mice expressing Cre recombinase under the control of a CD11c promoter (Jackson Laboratory). All mice were housed in the experimental animal center of the Fifth Affiliated Hospital of Sun Yat‐sen University. The use of animals was approved by the Animal Ethics Committee of the Fifth Affiliated Hospital of Sun Yat‐sen University (approval number: 00443).

### ALD‐DNA Immunization Induced Murine Lupus Models

The activated lymphocyte‐derived DNA (ALD‐DNA) was extracted from excessively activated lymphocytes and was characterized by immunogenicity. It can be recognized by the immune system, thereby triggering an immune response.^[^ ^73^ ^]^ The preparation of ALD‐DNA refers to the methods proposed by Xiong et al.^[^ ^24^ ^]^ Splenocytes were stimulated with Con A (5 µg mL^−1^) for 6 days to reach an apoptotic status. According to the instructions of the HiPure tissue DNA kit (Megan), genomic DNA was extracted from apoptotic splenocytes to obtain ALD‐DNA. The concentration of DNA was determined by the NanoDrop (Thermo Fisher Scientific).

Female 6–8 weeks old mice were subcutaneously injected under the dorsal skin with 0.1 ml of an emulsion containing 50 µg of ALD‐DNA plus complete Freund's adjuvant (CFA, Sigma–Aldrich), followed by two booster immunizations consisting of 50 µg DNA emulsified with incomplete Freund's adjuvant (IFA) at week 2 and 4. Blood and urine samples were collected every 2 weeks. All mice were sacrificed at week 12 after the first immunization. The spleen, lymph nodes, and kidneys were collected for further analysis.

### Drug Treatment in the Lupus Mice

According to the above method, the ALD‐DNA‐induced lupus model was established in WT and *Trem2* ^−/∐^ mice. After confirming the successful establishment of the murine lupus model, WT and *Trem2* ^−/−^ lupus mice were randomly assigned to receive either GSK484 hydrochloride (5 mg Kg^−1^/day), DNase I (2.5 mg Kg^−1^/day), ABAH (40 mg Kg^−1^/day), or an equal volume of PBS (control) via daily intraperitoneal injection for one month. The investigators performing the outcome assessments (sample collection and data analysis) were blinded to the group allocation. After one month of treatment, all mice were euthanized, and the blood, spleens, lymph nodes, and kidneys were isolated for further analysis.

### Isolation and Stimulation of Human DCs

Human CD11c^+^ DCs were isolated from peripheral PBMCs by magnetic bead separation (STEMCELL Technologies). The DCs (2 × 10^6^ cells mL^−1^) were incubated in complete RPMI 1640 medium with anti‐hTREM2 (MCE, PY314), or control IgG Abs (MCE) at 10 µg mL^−1^ for 1 h, and then activated with NETs from SLE patients overnight. The activation markers MHC II and CD86 of DCs were detected by flow cytometry.

### The Generation of DNA Immune Complexes

The total IgG from 5ml serum of lupus mice with a high titer of anti‐dsDNA Abs was isolated with a HiTrap Protein G affinity column (Cytiva). The concentration of IgG was detected by the bicinchoninic acid (BCA) protein assay kit (Thermo Fisher Scientific). Purified mouse ALD‐DNA (300µg mL^−1^) was mixed with IgG (1mg mL^−1^), and MPO (1mg mL^−1^, MCE) in 10µL of PBS. For BMDCs stimulation, complexes were diluted into 1mL of complete medium.

### Stimulation of BMDCs

The BMDCs were generated according to the above methods. WT and *Trem2* ^−/−^ BMDCs were respectively seeded in 6‐well plates and cultured in RPMI‐1640 supplemented with 10% FBS, 100 IU mL^−1^ penicillin, 100 µg mL^−1^ streptomycin. For stimulation, the WT and *Trem2* ^−/−^ BMDCs were treated with NETs or ICs for 15, 30, 60 min, 3, 6, and 12 h. And the control groups were treated with PBS.

### Phagocytosis Assays

To visualize the internalized NETs‐derived MPO, BMDCs were co‐cultured with NETs for 3 h. After washing with PBS, the BMDCs were fixed with 4% paraformaldehyde and permeabilized with 0.1% Triton X‐100. The cells were incubated with anti‐MPO rabbit antibody (EPR20257, Abcam) overnight. The following day, the cells were stained with an AF594 conjugated goat anti‐rabbit IgG (Abcam) to visualize the internalized MPO. Finally, cells were stained with green‐fluorescent phalloidin (MesGen) to visualize F‐actin and with Hoechst 33342 (Sigma‐Aldrich) to label nuclei. The BMDCs were observed under confocal microscopy.

In the flow cytometry‐detected phagocytosis experiments, WT and *Trem2* ^−/−^ BMDCs were pretreated with 5 µM Cytochalasin D (Cyto D) or vehicle DMSO for 30 mins. And then BMDCs were co‐cultured with 5µM PI (Thermo Fisher Scientific)‐labeled NETs for 3 h. At the end of the phagocytic phase, all cells in the wells were collected and washed, and then analyzed by flow cytometry.

To visualize the uptake of ALD‐DNA by BMDCs, ALD‐DNA was labeled with MFP488 using the Label IT^®^ Nucleic Acid Labeling Kit (Mirus), according to the standard protocol provided by the manufacturer. BMDCs were stimulated with DNA MFP488‐containing ICs for 4 h, washed, and analyzed by flow cytometry.

### Mass Spectrometry for the Analysis of NETs Proteins

The NETs from SLE patients were treated with DNase I (50 U mL^−1^, Roche) to detach DNA. NETs proteins were precipitated using 80% v/v acetone overnight at −20 °C. NETs protein (precipitated pellet) was obtained via centrifugation at 10 000 g for 30 min. Then, NETs protein was dissolved in the cell lysis buffer with protease inhibitor PMSF. NETs proteins were incubated with hTREM2‐Fc (R&D System) or control IgG1 (R&D System) and Protein A/G agarose beads (EMD Millipore) for a night at 4 °C with constant rotation. Beads were then washed five times using the cell lysis buffer. The beads bound to the antibody and the corresponding proteins were collected for analysis. Then, the mass spectrometry was performed on an Orbitrap Fusion Lumos using the parameters consistent with the previous protocol.^[^ ^74^ ^]^

### Surface Plasmon Resonance

Surface plasmon resonance (SPR) analysis was performed at 25 °C using a Biacore (Cytiva). Purified human TREM2 protein was immobilized onto a Biacore CM5 chip using the amine coupling kit (Cytiva) following the manufacturer's instructions. Purified human MPO was tested with a gradient concentration of 31.25, 62.5, 125, 250, 500, and 1000nm, respectively, and the response was measured in resonance units.

### Co‐IP Assays

For exogenous Co‐IP assays, the indicated plasmids were transfected into HEK293T cells (ATCC, CRL‐3216) using lipo2000 (Thermo Fisher Scientific) for 24 h, and cell lysates were immunoprecipitated with anti‐HA beads (Sigma–Aldrich) or anti‐FLAG beads (Sigma–Aldrich) and then assessed by Western blotting. For endogenous Co‐IP assays, the BMDC were stimulated with NETs or PBS for 24 h and then lysed to obtain cellular lysates. Protein from cell lysates was respectively incubated with anti‐mouse TREM2 Ab (clone EPR26210, Abcam) or isotype‐matched IgG and Protein A/G agarose beads (EMD Millipore) for a night at 4 °C with constant rotation. Beads were then washed five times using the cell lysis buffer, followed by Western blotting for TREM2 (clone EPR26210, Abcam), DAP12 (clone EPR24244‐76, Abcam), and SYK (clone D3Z1E, CST).

### RNA Sequencing

RNA sequencing was performed as previously described.^[^ ^75^ ^]^ Total RNA from whole blood cells of healthy controls and SLE patients was extracted using TRIzol reagent (Thermo Fisher) and was assessed for quantity and quality using a NanoDrop UV spectrophotometer and a Bioanalyser. The mRNA fragments were used for library construction. After passing the library check, the different libraries were sequenced in Illumina after pooling them according to the effective concentration and the target sequencing output data volume, yielding 150 bp paired‐end reads. Sequences were aligned to the reference human genome version GRCh38 using STAR. Gene expressions were obtained both as read counts directly from STAR as well as computed using RSEM in order to obtain gene and transcript level expression, FPKM values, for these stranded RNA libraries.

Sample clustering based on normalized log‐transformed FPKM values produces the hierarchy of samples. DESeq2 was used for differential gene expression analysis between two groups. P‐values lower than 0.05 and absolute log2 fold change greater than 1 were used as thresholds for significant differential expression. KEGG pathway enrichment analysis was performed based on the results of the rich factor calculation and hypergeometric test.

### Statistical Analyses

Data analyses were performed using GraphPad Prism 9.0 (GraphPad Software). The unpaired two‐tailed Student's *t*‐test was used for comparisons between two independent groups, while the paired two‐tailed Student's *t*‐test was used for matched samples. One‐way ANOVA with Tukey's post hoc test was used to analyze the differences among multiple groups. Pearson's correlation analysis was used for correlation analysis. Data are shown as the mean ± SEM. The P value of less than 0.05 was regarded as statistically significant.

## Conflict of Interest

The authors declare no conflict of interest.

## Author Contributions

J.S. and J.L. contributed equally to this work. X.H., Y.W., J.S., and J.L. conceived and designed the study. J.S., J.L., and L.Z. performed the majority of the experiments and data analysis. S.Z. and Y.X. assisted with experiments and provided scientific expertise. J.L., S.Z., and X.F. provided the human patient data and analysis. Y.W., J.S., J.L., and X.H. wrote the manuscript and modified the paper. All authors read the final version of the manuscript and approved the submission.

## Supporting information



Supporting Information

## Data Availability

All data associated with this study are present in the paper or the Supporting Information and are available from the corresponding author upon reasonable request.

## References

[1] M. Kiriakidou , C. L. Ching , Ann. Intern. Med. 2020, 172, Itc81. 32479157 10.7326/AITC202006020

[2] J. Tian , D. Zhang , X. Yao , Y. Huang , Q. Lu , Ann. Rheum. Dis. 2023, 82, 351. 36241363 10.1136/ard-2022-223035 PMC9933169

[3] G C. Tsokos , M S. Lo , P. C. Reis , K E. Sullivan , Nat. Rev. Rheumatology 2016, 12, 716. 27872476 10.1038/nrrheum.2016.186

[4] S. Lazar , J. M. Kahlenberg , Annu. Rev. Med. 2023, 74, 339. 35804480 10.1146/annurev-med-043021-032611

[5] M. K. Crow , Ann. Rheum. Dis. 2023, 82, 999. 36792346 10.1136/ard-2022-223741

[6] J. Dieker , J. Tel , E. Pieterse , A. Thielen , N. Rother , M. Bakker , J. Fransen , H B. P. M. Dijkman , J. H. Berden , J M. de Vries , L B. Hilbrands , J. van der Vlag , Arthritis Rheumatol. 2016, 68, 462. 26360137 10.1002/art.39417

[7] V. Papayannopoulos , Nat. Rev. Immunol. 2018, 18, 134. 28990587 10.1038/nri.2017.105

[8] S. Gupta , M. J. Kaplan , Nat. Rev. Nephrol. 2016, 12, 402. 27241241 10.1038/nrneph.2016.71 PMC5510606

[9] G S. Garcia‐Romo , S. Caielli , B. Vega , J. Connolly , F. Allantaz , Z. Xu , M. Punaro , J. Baisch , C. Guiducci , R L. Coffman , F J. Barrat , J. Banchereau , V. Pascual , Sci. Transl. Med. 2011, 3, 73. 10.1126/scitranslmed.3001201 PMC3143837 21389264

[10] V. Saferding , S. Blüml , J. autoimmunity 2020, 110, 102382. 31883831 10.1016/j.jaut.2019.102382

[11] J. Liu , X. Zhang , X. Cao , J. autoimmunity 2022, 132, 102856. 35773101 10.1016/j.jaut.2022.102856

[12] D. Ganguly , S. Haak , V. Sisirak , B. Reizis , Nat. Rev. Immunol. 2013, 13, 566. 23827956 10.1038/nri3477 PMC4160805

[13] A. Das , B A. Heesters , A. Bialas , J. O'Flynn , I R. Rifkin , J. Ochando , N. Mittereder , G. Carlesso , R. Herbst , M C. Carroll , Immunity 2017, 46, 106. 28099860 10.1016/j.immuni.2016.12.014 PMC8140609

[14] O. Takeuchi , S. Akira , Cell 2010, 140, 805. 20303872 10.1016/j.cell.2010.01.022

[15] T. Celhar , H. Yasuga , H. Y. Lee , O. Zharkova , S. Tripathi , S I. Thornhill , H K. Lu , B. Au , L H. K. Lim , T P. Thamboo , S. Akira , E K. Wakeland , J E. Connolly , A.‐M. Fairhurst , Arthritis Rheumatol. 2018, 70, 1597. 29687651 10.1002/art.40535 PMC6175219

[16] Y. Tsao , F. Tseng , C.‐W. Chao , M.‐H. Chen , Y. Yeh , B. O. Abdulkareem , S. Chen , W.‐T. Chuang , P.‐C. Chang , I.‐C. Chen , P.‐H. Wang , C.‐S. Wu , C.‐Y. Tsai , S.‐T. Chen , J. Clin. Invest. 2023, 133.

[17] J. Klesney‐Tait , I. R. Turnbull , M. Colonna , Nat. Immunol. 2006, 7, 1266. 17110943 10.1038/ni1411

[18] J. W. Ford , D. W. Mcvicar , Curr. Opin. Immunol. 2009, 21, 38. 19230638 10.1016/j.coi.2009.01.009 PMC2723941

[19] C.‐J. Liu , C.‐Y. Tsai , S.‐H. Chiang , S.‐J. Tang , N.‐J. Chen , T. W. Mak , G.‐H. Sun , K.‐H. Sun , J. autoimmunity 2017, 78, 92. 28089248 10.1016/j.jaut.2016.12.010

[20] M. Colonna , Nat. Rev. Immunol. 2023, 23, 580. 36750615 10.1038/s41577-023-00837-1 PMC9904274

[21] A. Bouchon , C. Hernández‐Munain , M. Cella , M. Colonna , J. Exp. Med. 2001, 194, 1111. 11602640 10.1084/jem.194.8.1111 PMC2193511

[22] C. Correale , M. Genua , S. Vetrano , E. Mazzini , C. Martinoli , A. Spinelli , V. Arena , L. Peyrin‐Biroulet , F. Caprioli , N. Passini , P. Panina‐Bordignon , A. Repici , A. Malesci , S. Rutella , M. Rescigno , S. Danese , Gastroenterology 2013, 144, 346. 23108068 10.1053/j.gastro.2012.10.040

[23] T. Nakao , Y. Ono , H. Dai , R. Nakano , A. Perez‐Gutierrez , G. Camirand , H. Huang , D A. Geller , A W. Thomson , Hepatology 2019, 70, 696. 30372546 10.1002/hep.30334 PMC6488456

[24] B. Qiao , J. Wu , Y. W. Chu , Y. Wang , D. P. Wang , H. S. Wu , S. D. Xiong , Rheumatology 2005, 44, 1108. 15840592 10.1093/rheumatology/keh656

[25] X. Zheng , Z. X Xiao , L. Hu , X. Fang , L. Luo , L. Chen , Cell Death Dis. 2019, 10, 393. 31113935 10.1038/s41419-019-1623-0 PMC6529467

[26] T. Möckel , F. Basta , J. Weinmann‐Menke , et al., Autoimmun Rev. 2021, 20, 102736. 33333233 10.1016/j.autrev.2020.102736

[27] E. C. Freitas , M. S. D. E. Oliveira , O. A. Monticielo , Clin. Rheumatol. 2017, 36, 2403. 28879482 10.1007/s10067-017-3811-6

[28] F. Furukawa , T. Yoshimasu , Autoimmun Rev. 2005, 4, 345. 16081025 10.1016/j.autrev.2005.01.006

[29] Z. X Xiao , X. Zheng , L. Hu , J. Wang , N. Olsen , S. G. Zheng , Front. immunol. 2017, 8, 1765. 29321778 10.3389/fimmu.2017.01765 PMC5732181

[30] Z. X Xiao , X. Hu , X. Zhang , Z. Chen , J. Wang , K. Jin , F. L. Cao , B. Sun , J A. Bellanti , N. Olsen , S. G. Zheng , Signal Transduction Targeted Ther. 2020, 5, 34. 10.1038/s41392-020-0139-5 PMC7145808 32296043

[31] M. F. Alarcon , Z. Mclaren , H. L. Wright , Front. immunology 2021, 12, 649693. 10.3389/fimmu.2021.649693 PMC7969658 33746988

[32] P. Yin , Z. Su , X. Shu , Z. Dong , Y. Tian , Int. Immunopharmacol. 2024, 143, 113286. 39378652 10.1016/j.intimp.2024.113286

[33] W. Lin , H. Chen , X. Chen , C. Guo , Antioxidants 2024, 13, 132. 38275657 10.3390/antiox13010132 PMC10812636

[34] T. K. Ulland , M. Colonna , Nat. Rev. Neurol. 2018, 14, 667. 30266932 10.1038/s41582-018-0072-1

[35] R. Lande , D. Ganguly , V. Facchinetti , L. Frasca , C. Conrad , J. Gregorio , S. Meller , G. Chamilos , R. Sebasigari , V. Riccieri , R. Bassett , H. Amuro , S. Fukuhara , T. Ito , Y.‐J. Liu , M. Gilliet , Sci. Transl. Med. 2011, 3, 73ra19. 10.1126/scitranslmed.3001180 PMC3399524 21389263

[36] H. Sha , Y. Liu , Y.‐L. Qiu , W.‐J. Zhong , N. Yang , C. Zhang , J. Duan , J.‐B. Xiong , C.‐X. Guan , Y. Zhou , Int. j.biol. sci. 2024, 20, 4713. 39309425 10.7150/ijbs.99456 PMC11414388

[37] F. Apel , L. Andreeva , L. S. Knackstedt , R. Streeck , C. K. Frese , C. Goosmann , K.‐P. Hopfner , A. Zychlinsky , Sci. Signaling 2021, 14, 673. 10.1126/scisignal.aax7942 33688080

[38] P.‐F. Ke , Y.‐T. Zhu , S.‐L. Cao , Y. Wang , S.‐T. Wu , Q.‐Q. He , L. Liang , J. Li , Int. j. clinical chem. 2024, 554, 117785. 10.1016/j.cca.2024.117785 38228224

[39] H. Lou , G. S. Ling , X. Cao , J. autoimmunity 2022, 132, 102861. 35872103 10.1016/j.jaut.2022.102861

[40] R. Furie , B H. Rovin , F. Houssiau , A. Malvar , Y. O Teng , G. Contreras , Z. Amoura , X. Yu , C.‐C. Mok , M B. Santiago , A. Saxena , Y. Green , B. Ji , C. Kleoudis , S W. Burriss , C. Barnett , D A. Roth , New England j. med. 2020, 383, 1117. 32937045 10.1056/NEJMoa2001180

[41] J. Liu , X. Zhang , Y. Cheng , X. Cao , Cell Mol. Immunol. 2021, 18, 2461. 34302064 10.1038/s41423-021-00726-4 PMC8298985

[42] J. Wang , J. Wang , W. Hong , L. Zhang , L. Song , Q. Shi , Y. Shao , G. Hao , C. Fang , Y. Qiu , L. Yang , Z. Yang , J. Wang , J. Cao , B. Yang , Q. He , Q. Weng , Nat. Commun. 2021, 12, 6198. 34707127 10.1038/s41467-021-26477-4 PMC8551263

[43] J. C. Crispín , M. I. Vargas‐Rojas , A. Monsiváis‐Urenda , et al., Clin. Immunol. 2012, 143, 45. 22239954 10.1016/j.clim.2011.12.004

[44] M P. Ashton , A. Eugster , S. Dietz , D. Loebel , A. Lindner , D. Kuehn , A E. Taranko , B. Heschel , A. Gavrisan , A.‐G. Ziegler , M. Aringer , E. Bonifacio , Arthritis Rheumatol. 2019, 71, 817. 30511817 10.1002/art.40793

[45] C. Gallo , E. Manzo , G. Barra , L. Fioretto , M. Ziaco , G. Nuzzo , G. d'Ippolito , F. Ferrera , P. Contini , D. Castiglia , C. Angelini , R. De Palma , A. Fontana , Cellular molecular life Sci. : CMLS 2022, 79, 369. 35723745 10.1007/s00018-022-04297-z PMC9207826

[46] A. Deczkowska , A. Weiner , I. Amit , Cell 2020, 181, 1207. 32531244 10.1016/j.cell.2020.05.003

[47] L. Zhong , Y. Xu , R. Zhuo , T. Wang , K. Wang , R. Huang , D. Wang , Y. Gao , Y. Zhu , X. Sheng , K. Chen , N. Wang , L. Zhu , D. Can , Y. Marten , M. Shinohara , C.‐C. Liu , D. Du , H. Sun , L. Wen , H. Xu , G. Bu , X.‐F. Chen , Nat. Commun. 2019, 10, 1365. 30911003 10.1038/s41467-019-09118-9 PMC6433910

[48] L. Piccio , C. Buonsanti , M. Cella , I. Tassi , R E. Schmidt , C. Fenoglio , J. Rinker , R T. Naismith , P. Panina‐Bordignon , N. Passini , D. Galimberti , E. Scarpini , M. Colonna , A H. Cross , Brain: j. neurol. 2008, 131, 3081. 10.1093/brain/awn217 PMC2577803 18790823

[49] C. Qin , M. Chen , M.‐H. Dong , S. Yang , H. Zhang , Y.‐F. You , L. Zhou , Y.‐H. Chu , Y. Tang , X.‐W. Pang , L.‐J. Wu , D.‐S. Tian , W. Wang , Brain: j. neurol. 2024, 147, 163. 10.1093/brain/awad321 37740498

[50] Y. Arinuma , Y. Hasegawa , T. Tanaka , Y. Matsueda , T. Wada , K. Oku , K. Yamaoka , Rheumatology 2023, 62, 105. 10.1093/rheumatology/keac488 36005852

[51] Y. Zhao , Q. Guo , J. Tian , W. Liu , X. Wang , Ageing Res. Rev. 2025, 103, 102596. 39608728 10.1016/j.arr.2024.102596

[52] S. Wang , M. Mustafa , C M. Yuede , S. V. Salazar , P. Kong , H. Long , M. Ward , O. Siddiqui , R. Paul , S. Gilfillan , A. Ibrahim , H. Rhinn , I. Tassi , A. Rosenthal , T. Schwabe , M. Colonna , J. Exp. Med. 2020, 217, 20200785. 10.1084/jem.20200785 PMC7478730 32579671

[53] C. Cochain , E. Vafadarnejad , P. Arampatzi , J. Pelisek , H. Winkels , K. Ley , D. Wolf , A.‐E. Saliba , A. Zernecke , Circ. Res. 2018, 122, 1661. 29545365 10.1161/CIRCRESAHA.117.312509

[54] A. Dabla , Y. C Liang , N. Rajabalee , C. Irwin , C G. J. Moonen , J V. Willis , S. Berton , J. Sun , mBio 2022, 13, 0145622. 10.1128/mbio.01456-22 PMC9426521 35924849

[55] S. Qu , S. Hu , H. Xu , Y. Wu , S. Ming , X. Zhan , C. Wang , X. Huang , Neuroscience bulletin 2024, 40, 17. 37498431 10.1007/s12264-023-01094-x PMC10774236

[56] A. B. Sigalov , Int. J. Mol. Sci. 2022, 23, 8857.36012120

[57] E. Iizasa , Y. Chuma , T. Uematsu , M. Kubota , H. Kawaguchi , M. Umemura , K. Toyonaga , H. Kiyohara , I. Yano , M. Colonna , M. Sugita , G. Matsuzaki , S. Yamasaki , H. Yoshida , H. Hara , Nat. Commun. 2021, 12, 2299. 33863908 10.1038/s41467-021-22620-3 PMC8052348

[58] E. Frangou , A. Chrysanthopoulou , A. Mitsios , K. Kambas , S. Arelaki , I. Angelidou , A. Arampatzioglou , H. Gakiopoulou , G. K. Bertsias , P. Verginis , K. Ritis , D. T. Boumpas , Ann. Rheum. Dis. 2019, 78, 238. 30563869 10.1136/annrheumdis-2018-213181 PMC6352428

[59] L P. Blanco , X. Wang , P M. Carlucci , J. J. Torres‐Ruiz , J. Romo‐Tena , H.‐W. Sun , M. Hafner , M J. Kaplan , Arthritis Rheumatol. 2021, 73, 2282. 33983685 10.1002/art.41796 PMC8589882

[60] C. Lood , L. P. Blanco , M. M. Purmalek , C. Carmona‐Rivera , S. S. De Ravin , C. K. Smith , H. L. Malech , J. A. Ledbetter , K. B. Elkon , M. J. Kaplan , Nat. Med. 2016, 22, 146. 26779811 10.1038/nm.4027 PMC4742415

[61] S. Sangaletti , C. Tripodo , C. Chiodoni , C. Guarnotta , B. Cappetti , P. Casalini , S. Piconese , M. Parenza , C. Guiducci , C. Vitali , M P. Colombo , Blood 2012, 120, 3007. 22932797 10.1182/blood-2012-03-416156

[62] W. Song , B. Hooli , K. Mullin , S. C. Jin , M. Cella , T K. Ulland , Y. Wang , R E. Tanzi , M. Colonna , J. Alzheimer's Association 2017, 13, 381. 10.1016/j.jalz.2016.07.004 PMC5299056 27520774

[63] C. L. Hartman , D. A. Ford , Arterioscler., Thromb., Vasc. Biol. 2018, 38, 1676. 30354198 10.1161/ATVBAHA.118.311427 PMC6324573

[64] L. K. Stamp , I. Khalilova , J. M. Tarr , R. Senthilmohan , R. Turner , R. C. Haigh , P. G. Winyard , A. J. Kettle , Rheumatology 2012, 51, 1796. 22814531 10.1093/rheumatology/kes193

[65] A. G. Siraki , Redox Biol. 2021, 46, 102109. 34455146 10.1016/j.redox.2021.102109 PMC8403760

[66] T. Reshetnyak , K. Nurbaeva , I. Ptashnik , A. Kudriaeva , A. Belogurov , A. Lila , E. Nasonov , Int. J. Mol. Sci. 2023, 24, 9210. 37298160 10.3390/ijms24119210 PMC10252548

[67] E. Villanueva , S. Yalavarthi , C. C. Berthier , J. B. Hodgin , R. Khandpur , A. M. Lin , C. J. Rubin , W. Zhao , S. H. Olsen , M. Klinker , D. Shealy , M. F. Denny , J. Plumas , L. Chaperot , M. Kretzler , A. T. Bruce , M. J. Kaplan , J. Immunol. 2011, 187, 538. 21613614 10.4049/jimmunol.1100450 PMC3119769

[68] M. Antonelou , E. Michaëlsson , R D. Evans , C. J. Wang , S R. Henderson , L S. Walker , R. J. Unwin , A D. Salama , JASN 2020, 31, 350. 31879336 10.1681/ASN.2019060618 PMC7003306

[69] K. Takahashi , C. D. Rochford , H. Neumann , J. Exp. Med. 2005, 201, 647. 15728241 10.1084/jem.20041611 PMC2213053

[70] A A. Nugent , K. Lin , B. van Lengerich , S. Lianoglou , L. Przybyla , S S. Davis , C. Llapashtica , J. Wang , D. J Kim , D. Xia , A. Lucas , S. Baskaran , P C. Haddick , M. Lenser , T K. Earr , J. Shi , J C. Dugas , B J. Andreone , T. Logan , H O. Solanoy , H. Chen , A. Srivastava , S B. Poda , P E. Sanchez , R J. Watts , T. Sandmann , G. Astarita , J W. Lewcock , K M. Monroe , G. Di Paolo , Neuron 2020, 105, 837. 31902528 10.1016/j.neuron.2019.12.007

[71] J. Chen , T. Wang , X. Li , L. Gao , K. Wang , M. Cheng , Z. Zeng , L. Chen , Y. Shen , F. Wen , Signal Transduction Targeted Ther. 2024, 9, 163. 10.1038/s41392-024-01881-6 PMC11180664 38880789

[72] O. O. Yeku , M. Barve , W. W. Tan , J. Wang , A. Patnaik , P. LoRusso , D. L. Richardson , A. R. Naqash , S. K. Lynam , S. Fu , M. Gordon , J. Hubbard , S. Kummar , C. Kyriakopoulos , A. Dowlati , M. Chamberlain , I. Winer , J. Immunotherapy Cancer 2025, 13, 010959. 10.1136/jitc-2024-010959 PMC11907075 40081941

[73] Z. K. Wen , W. Xu , L. Xu , Q. H. Cao , Y. Wang , Y. W. Chu , S. D. Xiong , Rheumatology 2007, 46, 1796. 18032537 10.1093/rheumatology/kem275

[74] F. He , T. Zhang , K. Xue , Z. Fang , G. Jiang , S. Huang , K. Li , Z. Gu , H. Shi , Z. Zhang , H. Zhu , L. Lin , J. Li , F. Xiao , H. Shan , R. Yan , X. Li , Z. Yan , Anal. Chim. Acta 2021, 1180, 338881. 34538334 10.1016/j.aca.2021.338881 PMC8310733

[75] A. Omer , M. C. Barrera , J L. Moran , X J. Lian , S. Di Marco , C. Beausejour , I.‐E. Gallouzi , Nat. Commun. 2020, 11, 4979. 33020468 10.1038/s41467-020-18734-9 PMC7536198

