# Supplementary material for: TREM2 Drives Neutrophil Extracellular Traps‐Induced Dendritic Cell Maturation and Contributes to Lupus Progression
Source: Adv Sci (Weinh). 2025 Nov 23;13(7):e08938. doi: 10.1002/advs.202508938 (PMC12866682; doi:10.1002/advs.202508938)
Supplement: Supplementary file 1 — Supporting Information [file ADVS-13-e08938-s001.docx]

TREM2 Drives Neutrophil Extracellular Traps-Induced Dendritic Cell Maturation and Contributes to Lupus Progression

**Authors:** *Jingxian Shu, Jiabi Liang, Linda Zeng, Shuping Zhong, Xueling Fang, Yating Xu, Yongjian Wu*, Xi Huang**

**Supplemental Materials and Methods**

**Pristane Induced Murine Lupus Models**: Female 6-8 weeks old mice were intraperitoneally (i.p.) injected once with 0.5 ml of pristane (Sigma-Aldrich). Mice were monitored for lupus manifestations until the end of the study. Blood and urine samples were collected every 4 weeks. All mice were sacrificed at week 32 after first immunization. The blood, spleen, lymph nodes and kidneys were collected for further analysis.

**BMDCs Immunization Induced Murine Lupus Models**: For BMDCs generation, bone marrow cells were isolated from the tibia and femur, pooled, and cultured in RPMI-1640 complete medium with 10% fetal bovine serum (Gibco) containing 20 ng/ml recombinant mouse (rm) GM-CSF (Pepro Tech) and 10 ng/ml rmIL-4 (Pepro Tech) for 6 days. For immunization, WT and *Trem2*^-/-^ BMDCs from CD45.2^+^ mice were incubated with 5 µg/ml ALD-DNA for 12 hours. After extensive wash, WT and *Trem2*^-/-^ BMDCs (5×10^5^) were intravenously (i.v.) injected to CD45.1^+^ mice respectively. To analysis the ratio of donor BMDCs in recipient mice, the recipient mice were euthanized at week 2 after BMDCs transfer. The spleens and lymph nodes were isolated, and the ratio of donor BMDCs (CD45.2^+^ CD11c^+^) were analyzed by flow cytometry. Blood and urine samples were collected every 4 weeks. To analyze the lupus-related manifestations, all recipient mice were sacrificed at week 20 after BMDCs transfer. The blood, spleen, lymph nodes and kidneys were collected for further analysis

**The Treatment Regimens of Lupus-prone MRL/Lpr Mice:** At 10 weeks old, 15 female MRL/Lpr mice were randomly assigned to one of three groups and received intraperitoneal injections until they reached 17 weeks old. The treatment groups included control IgG Abs (Merck, MABF1078) at a weekly dose of 50mg/Kg, anti-TREM2 Abs (Merck, MABN755) at a weekly dose of 50mg/Kg, or cyclophosphamide (CTX) (Sigma-Aldrich, C0768) at a weekly dose of 30 mg/kg. The investigators performing the outcome assessments (sample collection and data analysis) were blinded to the group allocation. Blood and urine samples were collected every 2 weeks. At 18 weeks old, all mice were euthanized, and the blood, spleens, lymph nodes and kidneys were isolated for further analysis.

**Kidney Histochemistry and Immunofluorescence Analysis:** The kidneys were removed, and fixed in formalin. Paraffin embedding, sectioning, hematoxylin and eosin staining, and Masson staining were performed. GN was scored by pathologist in a blinded manner. GN was scored on a scale of 1 to 6. 1, normal kidney. 2, mesangial expansion and increased mesangial cellularity with patent capillary loops. 3, enlarged glomeruli with moderate endocapillary cellularity. 4, three characteristics with marked endocapillary hypercellularity and loss of patency of most capillary loops. 5, few glomeruli with necrosis (karyorrhexis) or few active (cellular or fibrocellular) or organized (fibrous) crescents. 6, many active (cellular or fibrocellular) or organized (fibrous) crescents, necrosis (karyorrhexis), obliteration of glomerular architecture, segmental/global sclerosis). Severe disease was defined as a GN score of ≥4 and/or death before the experimental endpoint. Moderate disease was defined as a GN score of 2.5–3.5. Mild disease was defined as a GN score of 1-2.

To detect the deposition of IgG and C3 by immunofluorescence, kidneys were removed and immediately frozen in OCT. Kidney cryostat sections were stained with the following antibodies: rat anti-mouse C3 (Abcam), followed by AF594 labeled goat anti-rat IgG (Abcam), AF 488 goat anti-mouse IgG (Invitrogen) and DAPI (Beyotime). Images were captured with Zeiss 880 confocal microscope.

**Isolation and Stimulation of Mouse Bone Marrow Neutrophils:** Mouse bone marrow cells were harvested from the femurs and tibias. The neutrophils were isolated from mouse bone marrow cells by neutrophil extraction kit (Solarbio, P8550). 2 x 10^6^ neutrophils were seeded in 6-well plates. For NETs formation, neutrophils were stimulated with PMA (50nM) for 4 h. Supernatants were removed and then ice-cold PBS was added to petri dish. NETs-containing supernatants were vigorously collected and centrifuged at 450g for 10 minutes at 4 °C to obtain cell-free supernatants. Then the supernatant was centrifuged at 18,000 g for 15 min at 4 °C. The bottom product was collected and re-suspended with ice-cold PBS.

**Enzyme-Linked Immunosorbent Assays (ELISAs):** To detect the concentration of human MPO-DNA complex, CitH3 and NE, we used the commercially available ELISAs kits (Jiangsu Meimian Industrial Co., Ltd) to measure its levels in plasma obtained from healthy controls and SLE patients. Plasma was isolated from whole blood using lithium heparin tubes and centrifuge at 3000 r.p.m for 10 min. Plasma were collected and initially diluted to 1:5. And the detection procedures were according to manufacturer’s instructions.

To detect the concentration of mice anti-dsDNA abs, serum was collected by retro-orbital venous plexus. Mice urine samples were collected at indicated timepoint. According to instructions of ELISAs kit (Jiangsu Meimian Industrial Co., Ltd), we detect the serum anti-dsDNA Abs, BAFF levels and urine albumin levels.

**Flow Cytometry Analysis:** The cells were resuspended in ice-cold PBS containing 1% BSA and were stained with the fluorescent dye-labeled Abs. The following fluorescent dye–labeled anti-human Abs were used: CD3 (clone HIT3a, BioLegend); CD4 (clone OKT4, BioLegend); CD19 (clone HIB19, BioLegend); CD11c (clone B-ly6, BD); CD14 (clone M5E2, BioLegend); Lineage cocktail (clone UCHT1, HCD14, 3G8, HIB19, 2H7, HCD56, BioLegend); HLA-DR (clone L243, BioLegend); CD86 (clone BU63, BioLegend); CD123 (clone 6H6, BioLegend); TREM2 (clone 237920, R&D Systems). The following fluorescent dye–labeled anti-mouse Abs were used: CD3 (clone 17A2, BioLegend); CD4 (clone GK1.5, BioLegend); CD8 (clone 53-6.7, BioLegend); CD19 (clone 6D5, BioLegend); CD138 (clone 281-2, BioLegend); B220 (clone RA3-6B2, BioLegend); PD-1 (clone EH12.2H7, BioLegend); CXCR5 (clone L138D7, BioLegend); CD11b (clone M1/70, BioLegend); Ly6G (clone 1A8, BioLegend); CD11c (clone N418, BioLegend); F4/80 (clone BM8, BioLegend); Fas (clone SA367H8, BioLegend); PNA (clone MECA-79, BioLegend); IgD (clone 11-26c.2a, BioLegend); CD86 (clone GL-1, BioLegend); MHC Ⅱ (clone M5/114.15.2, BioLegend); CCR7 (clone 4B12, BioLegend); CD62L (clone QA18A74, BioLegend); IFNγ (clone 2E2, BioLegend); IL-4 (clone 11B11, BioLegend); IL-17 (clone 9B10, BioLegend); CD45.2 (clone 104, BioLegend). Samples were analyzed on a Attune NxT flow cytometer (Thermo Fisher Scientific) with Attune NxT version 4.2 software, and analyzed with FlowJo version 10.8.1 software (Tree Star).

**Spleen Cell Proliferation Assays:** WT versus *Trem2*^-/-^ BMDCs were induced from bone marrow cells of the indicated genotype mice by the above methods, and pretreated with NETs for 24h. Spleen cells isolated from WT mouse were labeled with 5μM CFSE (Invitrogen). The CFSE-labeled spleen cells (1 x 10^5^ cells / well) were cultured in U-type 96-well plate with WT or *Trem2*^-/-^ BMDCs (1 x 10^5^ cells / well). CFSE dilution of CD4^+^ T and B cells was analyzed by flow cytometry on day 3.

**Solid-Phase Binding:** Solid-phase binding was performed in a 96-well plate using enzyme-linked immunosorbent assay (ELISA). WT, R47H, and T96K TREM2 proteins in PBS were respectively immobilized onto plate wells overnight with 100 mL per well. After the plates were washed three times with 0.1% PBST (PBS with 0.1% Tween 20) and blocked with 4% BSA for 1 h, purified MPO protein (0-120 nM) diluted in PBS was added and incubated for 1 h. After washing, the bound proteins were detected with Rabbit MPO antibody (Abcam, ab208670, 1:5000) for 1 h. After primary antibody incubation, plates were washed and then incubated with HRP conjugated anti-Rabbit – IgG for 30 min. Finally, plates were washed again and developed with TMB and the absorbance at 450 nm was recorded.

**Plasmid construction:** Plasmids containing human TREM2, MPO, CTSG or AZU1 with an C-terminal tag were constructed. Full length genes were amplified by reverse transcription-PCR and cloned into pcDNA3.1(+) vector. Pro-peptide (Pro), light chain (L), heavy chain (H) and Pro-peptide + light chain (Pro+L) domains of MPO were cloned based on the full-length MPO plasmid. TREM2 plasmids deleting transmembrane domain (ΔTM), or cytoplasmic domain (ΔCD), or extracellular domain (ΔIg) were constructed on the basis of full-length TREM2 plasmid. R47H, R62H, T96K, H157Y of TREM2 were generated with MultiS Fast Mutagenesis kit V2 (Cat. #C215-01, Vazyme, Nanjing, China) following the manufacturer’s recommendations. All constructed plasmids were confirmed by sequencing and their expressions in 293T cells were detected by western blot with anti-Tag antibodies.

**RT-PCR:** Total RNA was extracted from BMDCs using TRIzol (Invitrogen) reagent according to the manufacturer’s protocol, and the concentration of total RNA was quantified using NanoDrop (Thermo Fisher Scientific). Full-length cDNAs were synthesized using the RevertAid First Strand cDNA Synthesis Kit (Thermo Fisher Scientific). RT-PCR was carried out using cDNA, SYBR green PCR master mix (Applied Biosystems, Foster City, CA, USA), forward and reverse primers for IFNα, IFNβ, BAFF, IL-4, IL-6, IL-10 and IL-12 gene on a real-time PCR system (CFX96, Bio-Rad Laboratories). Relative gene expression was normalized against β-actin, and fold change in mRNA expression was determined using the ΔΔCt method. Primers used in these experiments are shown in Supplemental table 3.

**Western blot:** Cells were lysed in cell lysis buffer with protease inhibitor phenylmethylsulfonyl fluoride (PMSF) for 30 min on ice. The concentration of protein samples was determined by the BCA protein assay kit (Thermo Fisher Scientific). Protein samples were heated with loading buffer at 100°C for 10 mins. Equal amounts of protein samples were loaded onto 10% or 12.5% SDS-PAGE gel and transferred to PVDF membranes. Membranes were blocked with TBST containing 5% milk, followed by incubation with anti-SYK (clone D3Z1E, CST), anti-p-SYK (clone 65E4, CST), anti-ERK1/2 (clone 137F5, CST), anti-p-ERK1/2 (clone D13.14.4E, CST), anti-NF-Κb (clone D14E12, CST), anti-p-NF-κB (clone 93H1, CST), anti-IRF3 (clone EPR2418Y, Abcam), anti-p-IRF3 (clone 4D4G, CST), anti-TBK1 (clone D1B4, CST), anti-TBK1 (clone D52C2, CST), anti-HA (clone 6E2, CST), ant-FLAG (clone D6W5B, CST).

**Protein Structure Prediction and Visualization:** The protein sequences of TREM2 (accession number: Q9NZC2) and MPO (accession number: P05164) were retrieved from the UniProt database. The corresponding protein sequences were then input into the AlphaFold3 protein structure prediction program to obtain the protein conformations after interaction ^1^. The protein complex conformation with the highest score was selected and subjected to annotation and visualization using PyMOL software (version 3.0.3) ^2^. The binding free energy (ΔG) of the interacting proteins was calculated using the PDBePISA online program.

(https://www.ebi.ac.uk/msd-srv/prot_int/pistart.html)

**References:**

**1.** Abramson J, Adler J, Dunger J, et al. Accurate structure prediction of biomolecular interactions with AlphaFold 3. *Nature.* Jun 2024;630(8016):493-500.

**2.** Makarewicz T, Kaźmierkiewicz R. Molecular dynamics simulation by GROMACS using GUI plugin for PyMOL. *Journal of chemical information and modeling.* May 24 2013;53(5):1229-1234.

**Supplemental Figures**


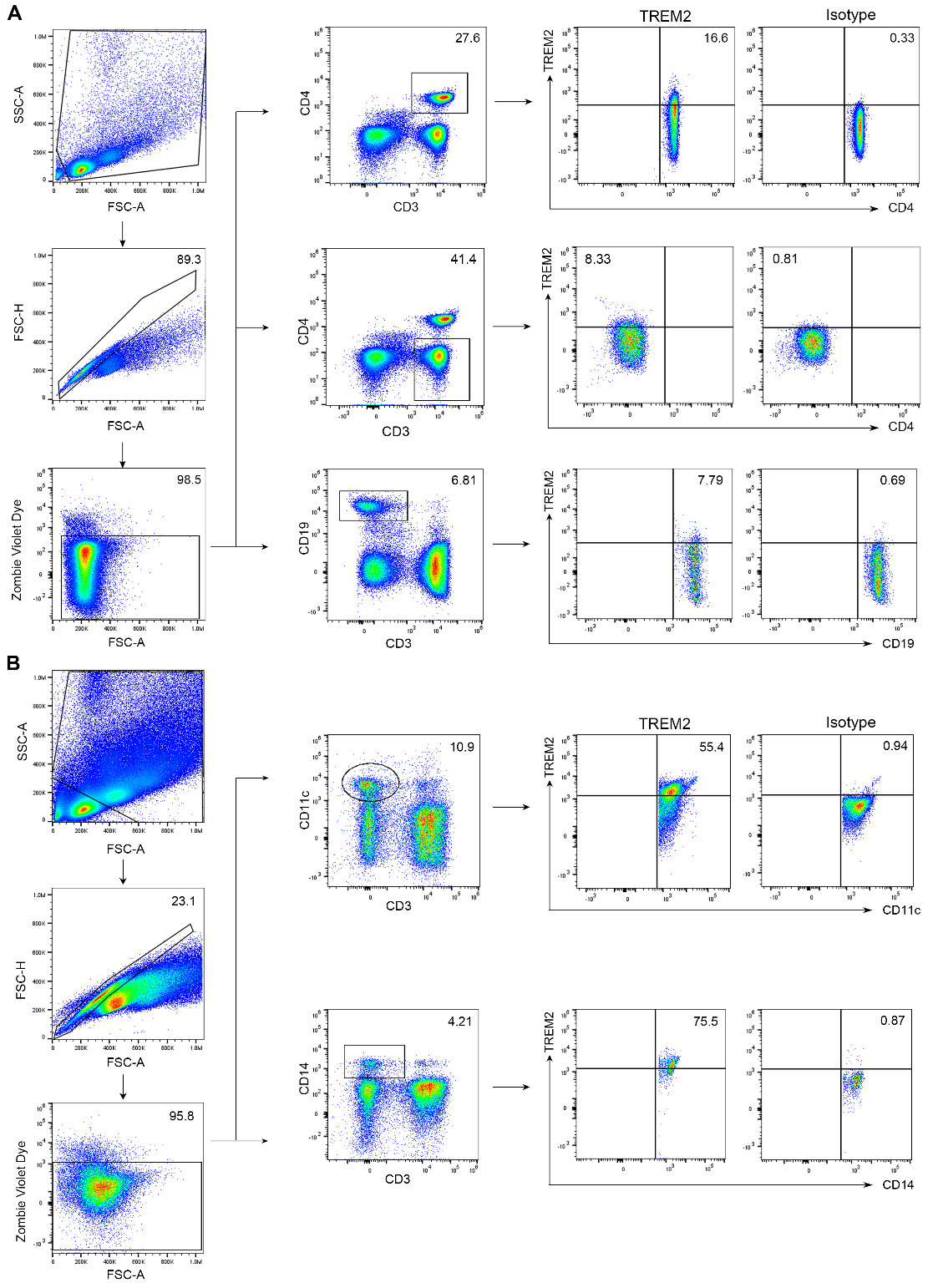


**Figure S1. related to Figure 1. Gating strategies in human PBMCs**. PBMCs were gated as single, live (Zombie Violet dye) cells. (A) The gating strategies of lymphocytes. CD4^+^ T cells were gated as CD3^+^ CD4^+^ cells. CD8^+^ T cells were gated as CD3^+^ CD4^-^ cells. B cells were gated as CD3^-^ CD19^+^ cells. (B) The gating strategies of myeloid cells. Dendritic cells (DCs) were gated as CD11b^+^ CD11c^+^ cells. Monocytes were gated as CD11b^+^ CD14^+^ cells. IgG isotype was used to set the cutoff value of TREM2^+^ cells and TREM2^-^ cells.


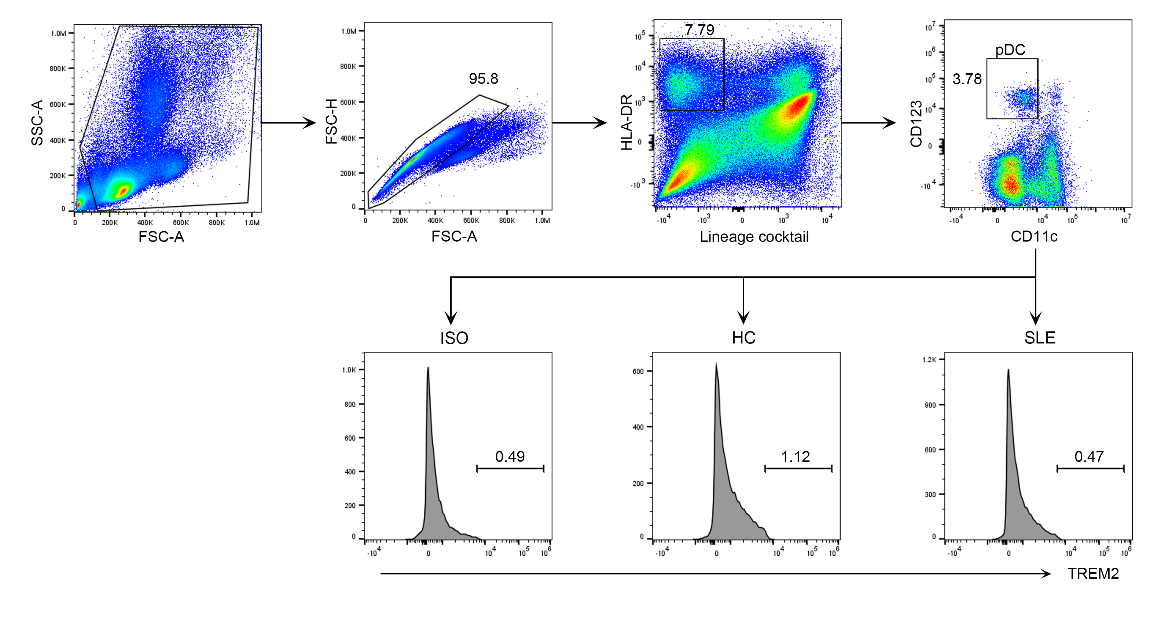


**Figure S2. related to Figure 1. TREM2 expression on plasmacytoid DCs (pDCs) was detected in healthy controls and SLE patients.** pDCs were gated as Lineage cocktail^-^ HLA-DR^+^ CD11c^-^ CD123^+^ cells. TREM2 expression was hardly detected on pDCs from HCs and SLE patients. IgG isotype was used to set the cutoff value of TREM2^+^ cells and TREM2^-^ cells.


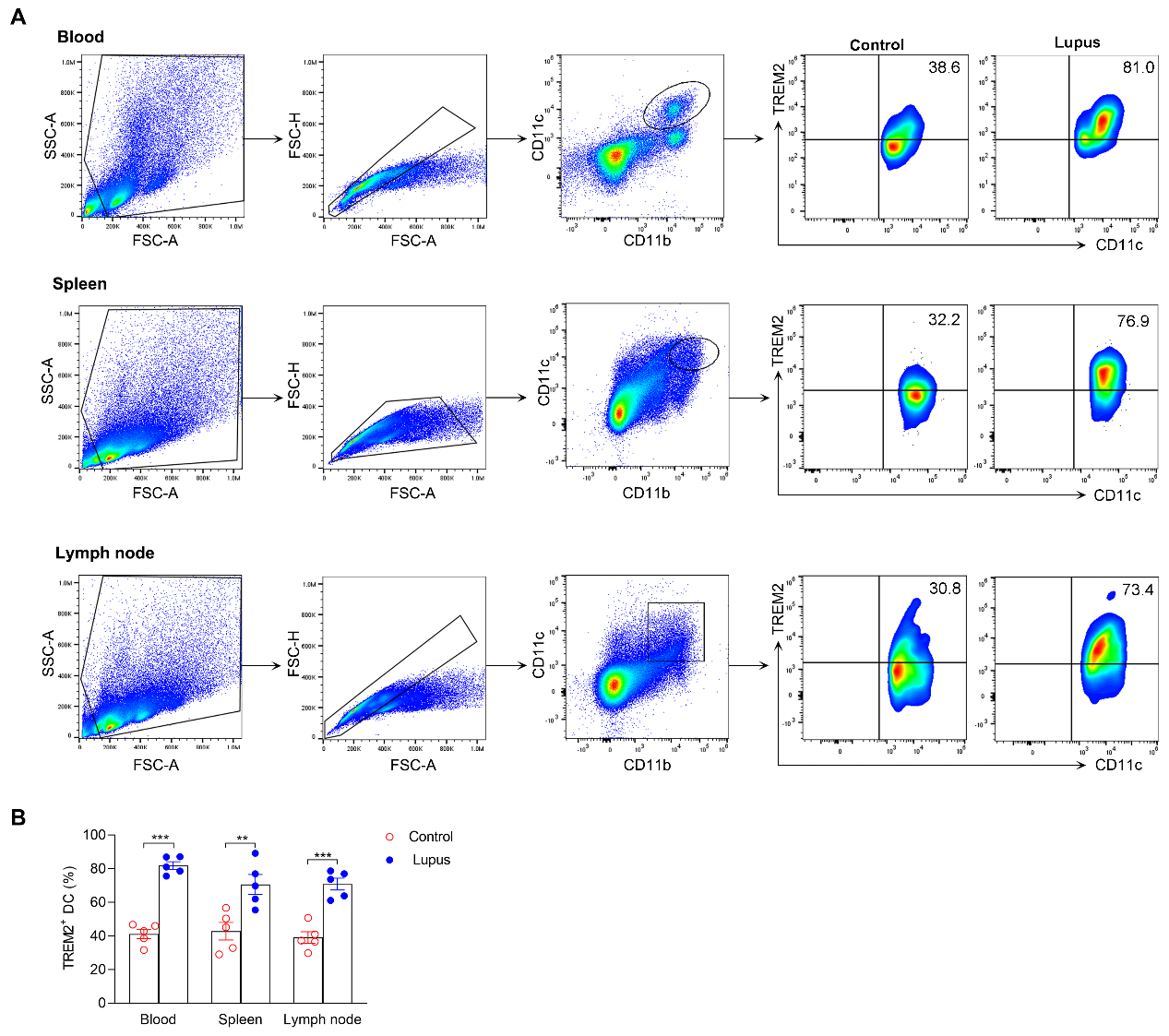


**Figure S3. related to Figure 1. TREM2 expression is upregulated on DCs from lupus mice. (A)** Gating strategies in mice. TREM2 expressions on DCs (Gated as CD11b^+^ CD11c^+^ cells) from blood, spleen and lymph node were determined by flow cytometry. **(B)** Quantification of TREM2 expression on DCs in lupus mice (n=5, unpaired 2-tailed Student’s t test). The results are presented as the mean ± SEM from three independent experiments. *, P < 0.05; **, P < 0.01; ***, P < 0.001.


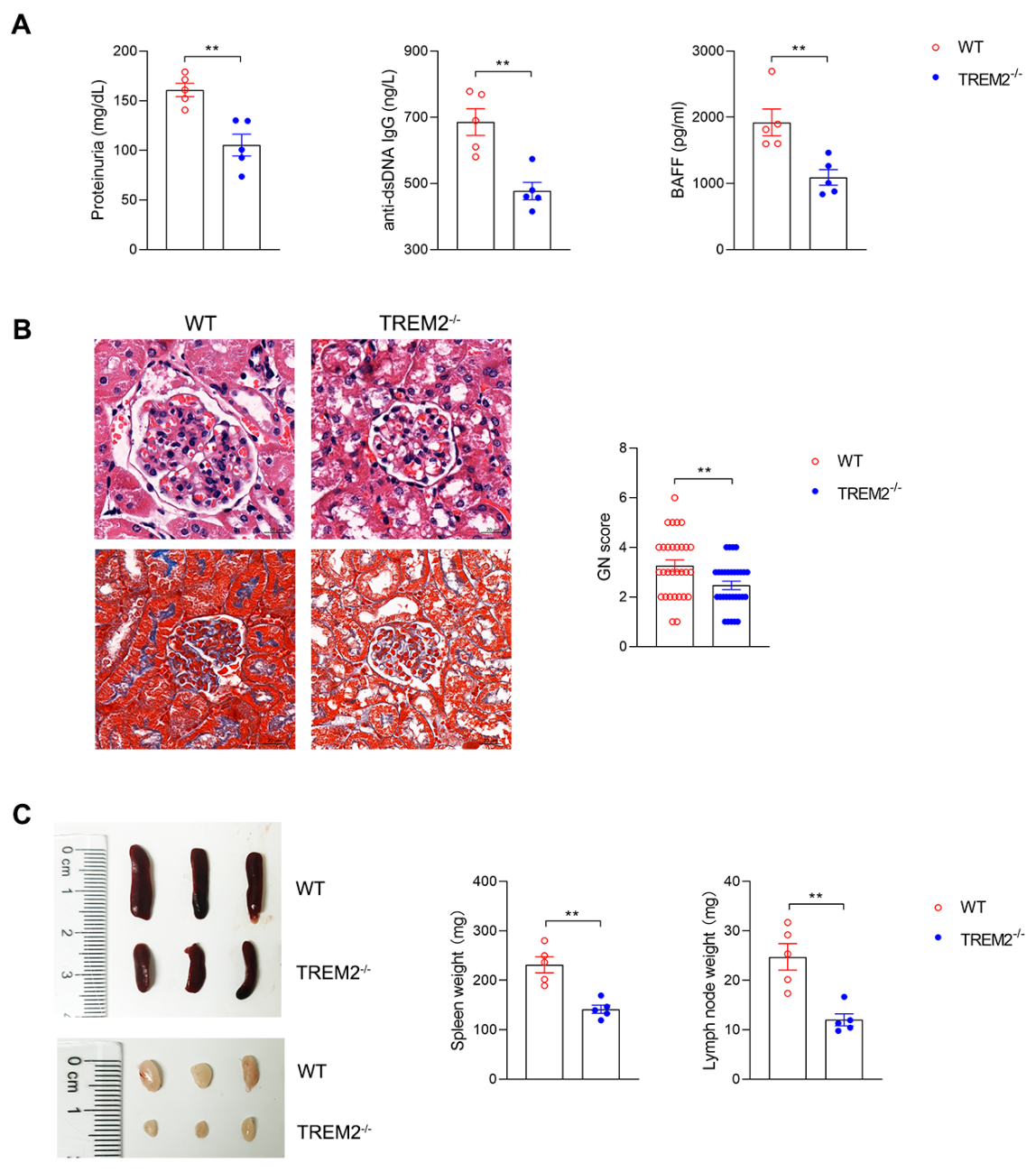


**Figure S4. related to Figure 2. *Trem2* deficiency attenuates lupus manifestation in the pristane induced murine lupus model. A-C,** pristane induced lupus model was established in WT and *Trem2*^-/-^ mice. **(A)** The levels of proteinuria, serum anti-dsDNA Abs and BAFF were detected by ELISA at 6 months after pristane injection (n=5, unpaired 2-tailed Student’s t test). **(B)** Renal damage was evaluated by H&E and Masson staining, and scored with glomerulonephritis (GN) scores (n=30, unpaired 2-tailed Student’s t test). Scale bars, 50 μm. **(C)** The gross appearance and the weight of spleens or lymph nodes were recorded (n=5, unpaired 2-tailed Student’s t test). The results are presented as the mean ± SEM from three independent experiments. *, P < 0.05; **, P < 0.01; ***, P < 0.001.


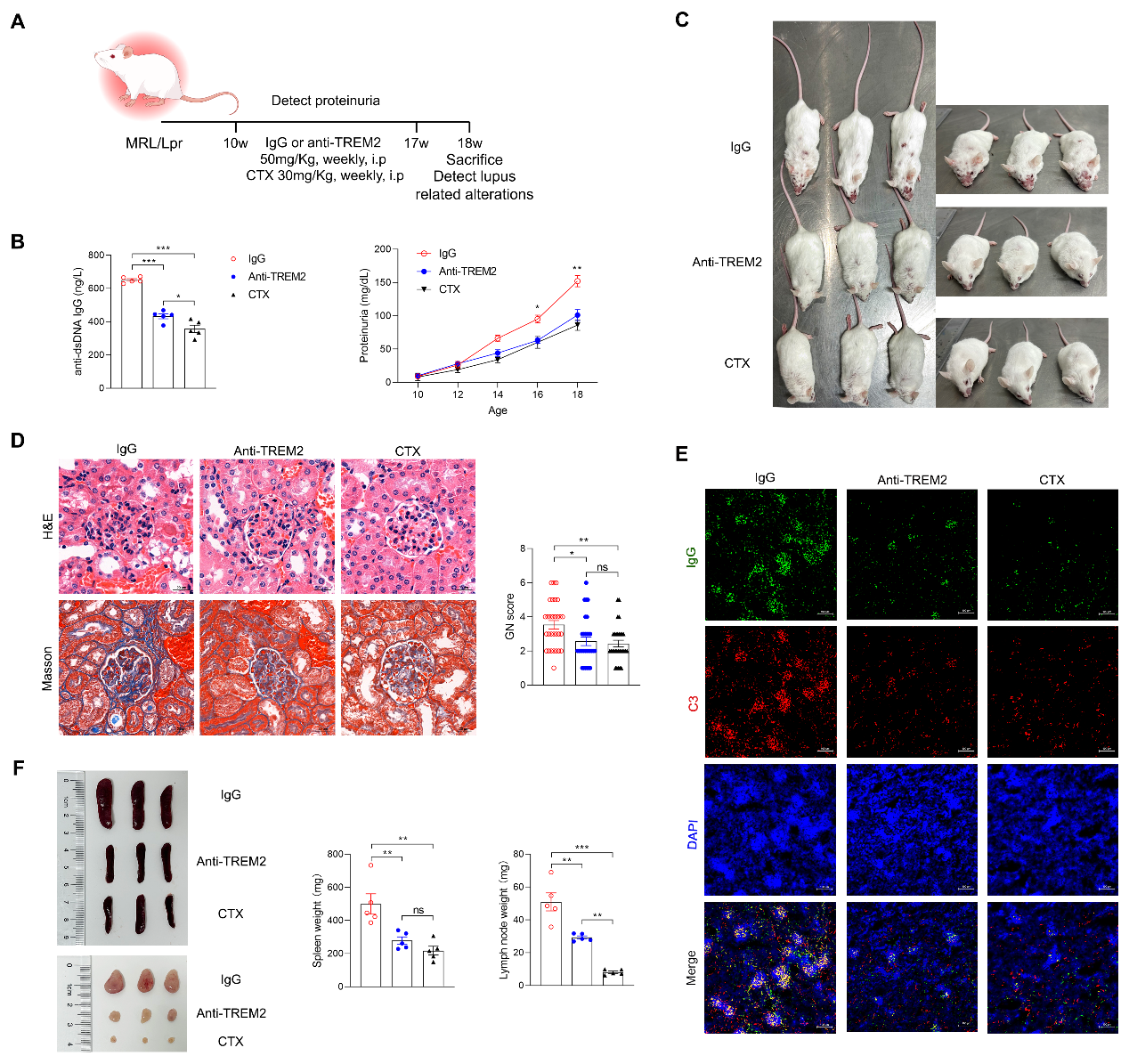


**Figure S5. related to Figure 2. TREM2 blocking alleviates lupus manifestation in the lupus-prone MRL/Lpr mice.** **(A)** The MRL/Lpr mice were randomly divided into three groups, which were respectively treated with TREM2 blocking Ab (anti-TREM2), cyclophosphamide (CTX) and control IgG Ab. **(B)** The levels of proteinuria and serum anti-dsDNA Abs were detected by ELISA (n=5, One-way ANOVA). **(C)** The lupus-like facial skin lesions of MRL/Lpr mice in each group were recorded at 16 weeks of age. **(D)** Renal damage was evaluated by H&E and Masson staining, and scored with GN scores (n=30, One-way ANOVA). Scale bars, 50 μm. **(E)** Immunofluorescence assay was used to analyze the deposition of IgG and C3 in renal sections. Scale bar, 100 μm. **(F)** The gross appearance and the weight of spleens or lymph nodes were recorded at 18 weeks of age (n=5, One-way ANOVA). The results are presented as the mean ± SEM from three independent experiments. *, P < 0.05; **, P < 0.01; ***, P < 0.001.


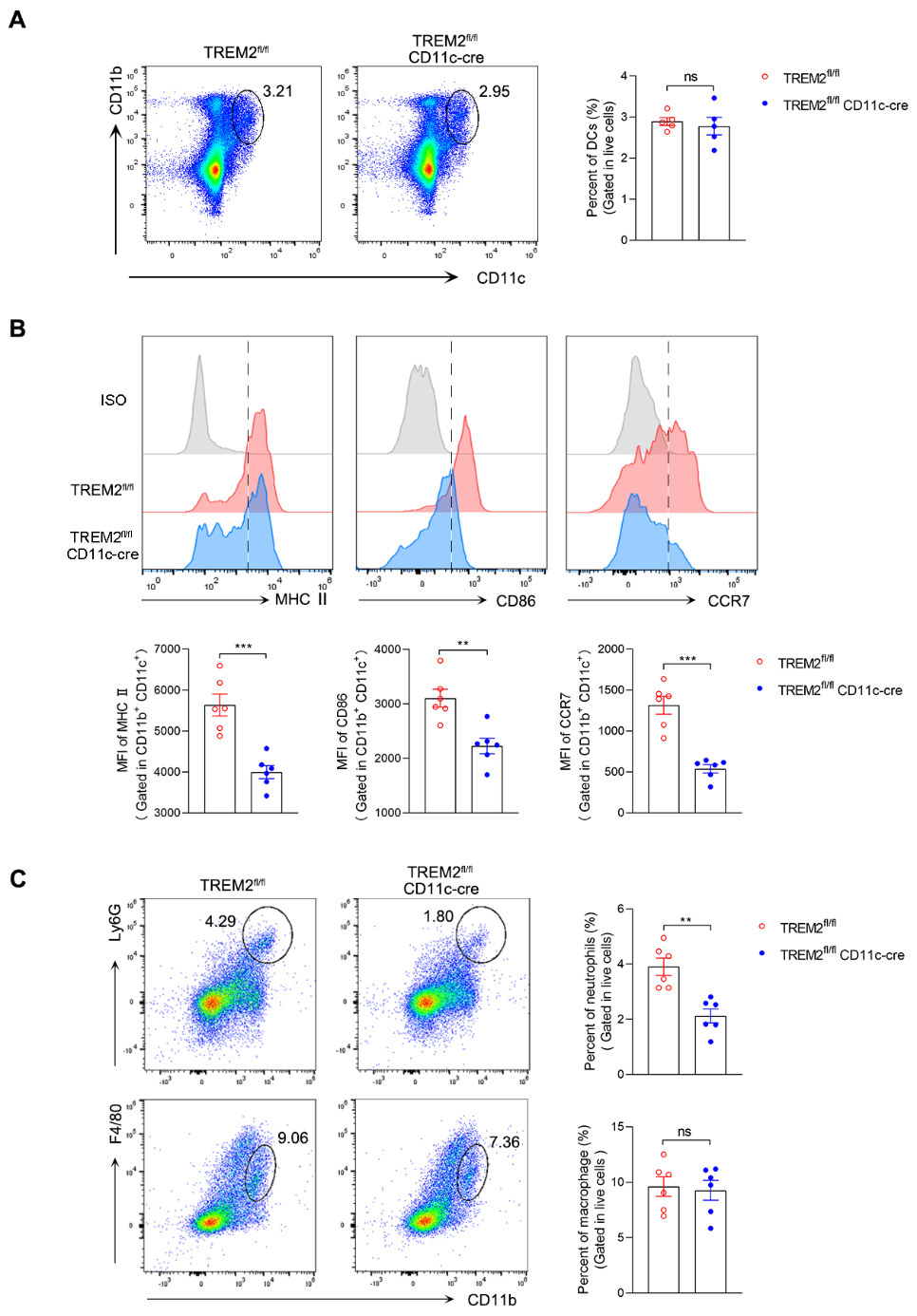


**Figure S6. related to Figure 2.** ***Trem2* knockout in DCs alleviates the inflammatory response of myeloid cells in lupus mice.** ALD-DNA induced lupus model was established in WT and *Trem2*^-/-^ mice. **(A)** The ratio of spleen DCs (CD11b^+^ CD11c^+^) was detected by flow cytometry (n=6, unpaired 2-tailed Student’s t test). **(B)** The activation marker of spleen DCs, MHC Ⅱ, CD86, CCR7, was detected by flow cytometry (n=6, unpaired 2-tailed Student’s t test). **(C)** The ratio of spleen neutrophils (CD11b^+^ Ly6G^+^) and macrophage (CD11b^+^ F4/80^+^) was detected by flow cytometry (n=6, unpaired 2-tailed Student’s t test). The results are presented as the mean ± SEM from three independent experiments. *, P < 0.05; **, P < 0.01; ***, P < 0.001; ns, no significance difference.


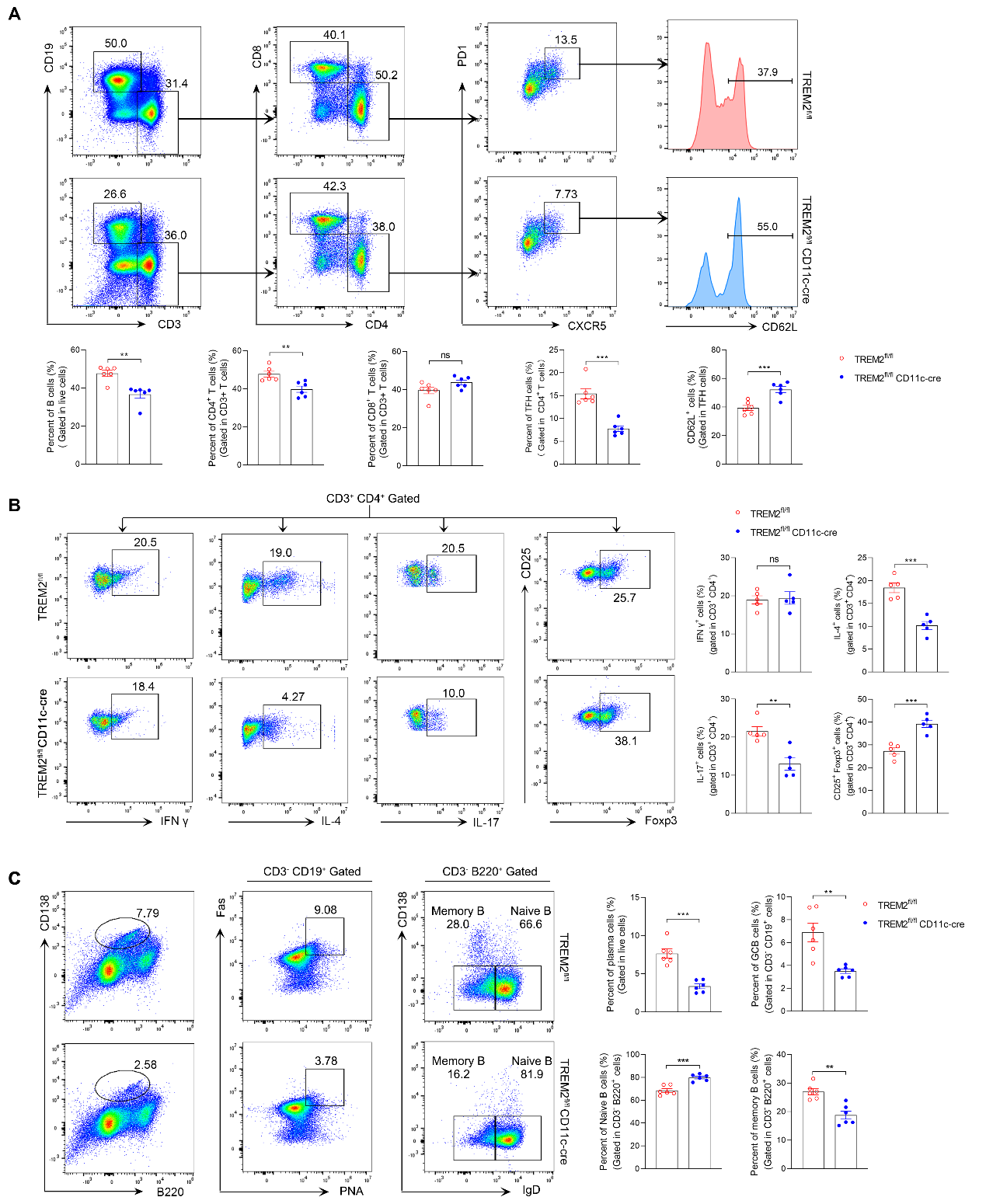


**Figure** **S7. related to Figure 2. *Trem2* knockout in DCs alleviates the inflammatory response of lymphocytes in lupus mice.** **(A)** Flow cytometry was used to analyze the frequencies of B cells (CD3^-^ CD19^+^), CD4^+^ T cells (CD3^+^ CD4^+^), CD8^+^ T cells (CD3^+^ CD8^+^), TFH cells (CD3^+^ CD4^+^ CXCR5^+^ PD1^+^), and the inactivation marker CD62L in TFH (n=6, unpaired 2-tailed Student’s t test). **(B)** Th1 cells (IFNγ^+^ CD4^+^ T), Th2 cells (IL-4^+^ CD4^+^ T), Th17 cells (IL-17^+^ CD4^+^ T), Treg cells (CD25^+^ Foxp3^+^ CD4^+^ T) were analyzed by flow cytometry (n=6, unpaired 2-tailed Student’s t test). **(C)** Flow cytometry was used to analyze the ratios of plasma cells (CD3^-^ CD138^+^ B220^med^), GCB cells (CD3^-^ CD19^+^ PNA^+^ Fas^+^), naïve B (CD3^-^ B220^+^ IgD^+^ CD138^-^), memory B(CD3^-^ B220^+^ IgD^-^ CD138^-^) between *Trem2*^fl/fl^ and *Trem2*^fl/fl^ CD11c-cre lupus mice (n=6, unpaired 2-tailed Student’s t test). The results are displayed as the mean ± SEM from three independent experiments. ns indicates no significance, *P < 0.05, **P < 0.01, ***P < 0.001.


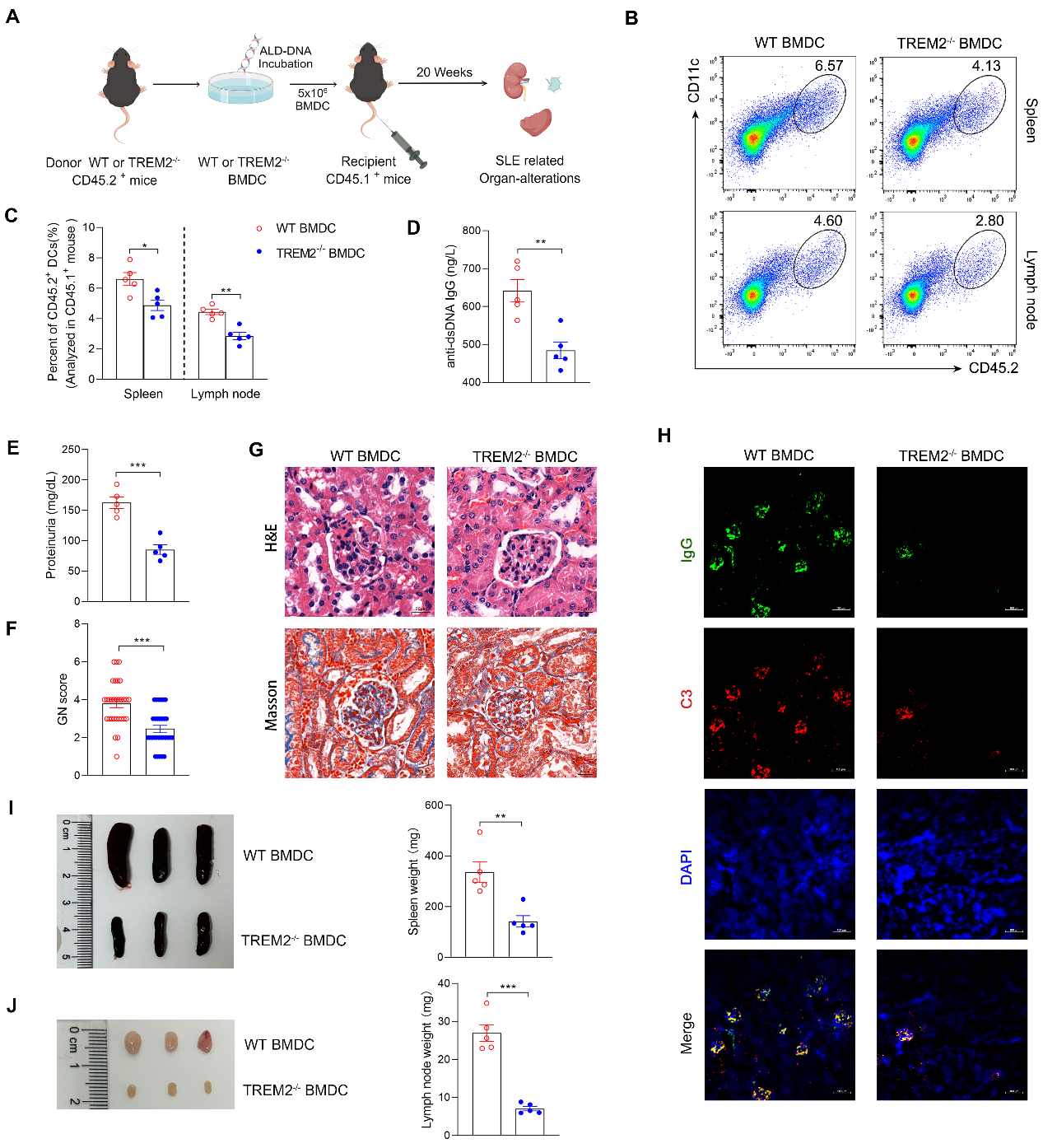


**Figure S8. related to Figure 2. Lupus mice induced by BMDCs with *Trem2* deficiency exhibits alleviated manifestations.** **(A)** The construction of BMDCs induces murine lupus model. CD45.2^+^ mice-derived WT or *Trem2*^-/-^ BMDCs were incubated with ALD-DNA, and then injected into CD45.1^+^ mice respectively. **(B-C)** At weeks 2 after BMDCs injection, the spleens and lymph node were harvested and the percentage of donor DCs (CD45.2^+^ CD11c^+^) were analyzed by flow cytometry (n=5, unpaired 2-tailed Student’s t test). **(D-E)** The serum and urine samples from each mouse were collected at weeks 20 after the transfer of donor DCs. The levels of anti-dsDNA Ab and proteinuria were detected by ELISA (n=5, unpaired 2-tailed Student’s t test). **(F-G)** The paraffin-embedded kidney sections were stained with H&E and Masson. The Kidney pathology was evaluated by GN scores. Scale bar, 20 μm (n=30, unpaired 2-tailed Student’s t test). **(H)** Immunofluorescence assays were used to analyze the deposition of IgG and C3 in renal sections from CD45.1^+^ recipient mice; Scale bar, 100 μm. **(I-J)** The gross appearance and the weight of spleens or lymph nodes from mice of the indicated groups were recorded at weeks 20 (n=5, unpaired 2-tailed Student’s t test). The results are presented as the mean ± SEM from three separate experiments. *, P < 0.05; **, P < 0.01; ***, P < 0.001.


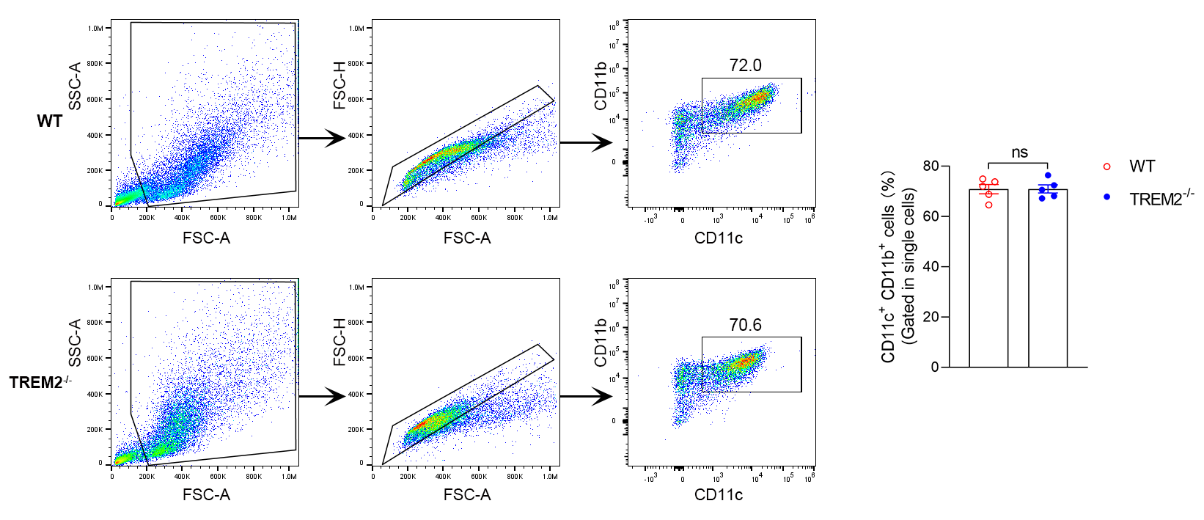


**Figure S9. related to Figure 4. The ratio of BMDCs induced from bone marrow cells of WT and *Trem2*^-/-^ mice.** The bone marrow cells from WT and *Trem2*^-/-^ mice were collected and induced to BMDCs in the culture with GM-CSF (20 ng/ml) and IL-4 (10 ng/ml) for 6 days, respectively. The flow cytometry was used to analyze the ratio of BMDC (CD11c^+^ CD11b^+^) in single cultured cells (n=5, unpaired 2-tailed Student’s t test). The results are presented as the mean ± SEM from three independent experiments. ns, no significance.


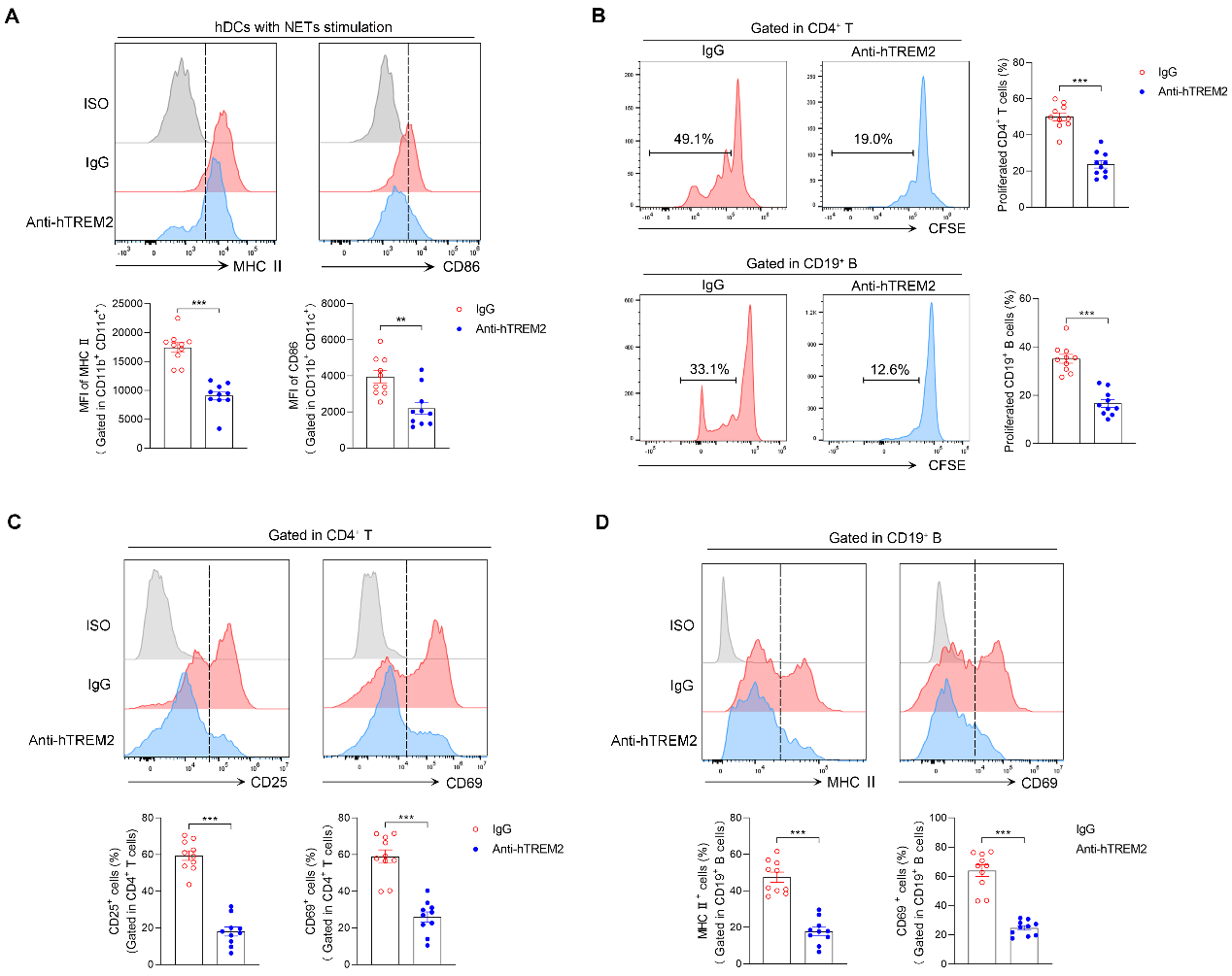


**Figure S10. related to Figure 4. Blockade of TREM2 impairs NETs-induced maturation and antigen presentation ability in human DCs.** **(A)** The DCs isolated from peripheral blood of SLE patients were respectively pretreated with hTREM2 blocking Ab (anti-hTREM2), or control IgG Ab, and then stimulated with NETs for 24 hours. The maturation markers (MHC Ⅱ and CD86) of BMDCs from two groups were analyzed by flow cytometry (n=10, paired 2-tailed Student’s t test). **(B)** NETs-stimulated, anti-hTREM2/IgG-treated DCs were cocultured with CFSE-labeled human PBMCs for 3 days. Proliferation of CD4^+^ T cells (CD3^+^CD4^+^) and B cells (CD3^-^CD19^+^) was assessed by flow cytometry based on CFSE dilution (n=10, paired 2-tailed Student’s t test). **(C-D)** The expression of activation markers on CD4^+^ T cells (CD25 and CD69) and B cells (MHC II and CD69) was analyzed by flow cytometry after coculture (n=10, paired 2-tailed Student’s t test). The results are presented as the mean ± SEM from three independent experiments. *, P < 0.05; **, P < 0.01; ***, P < 0.001.


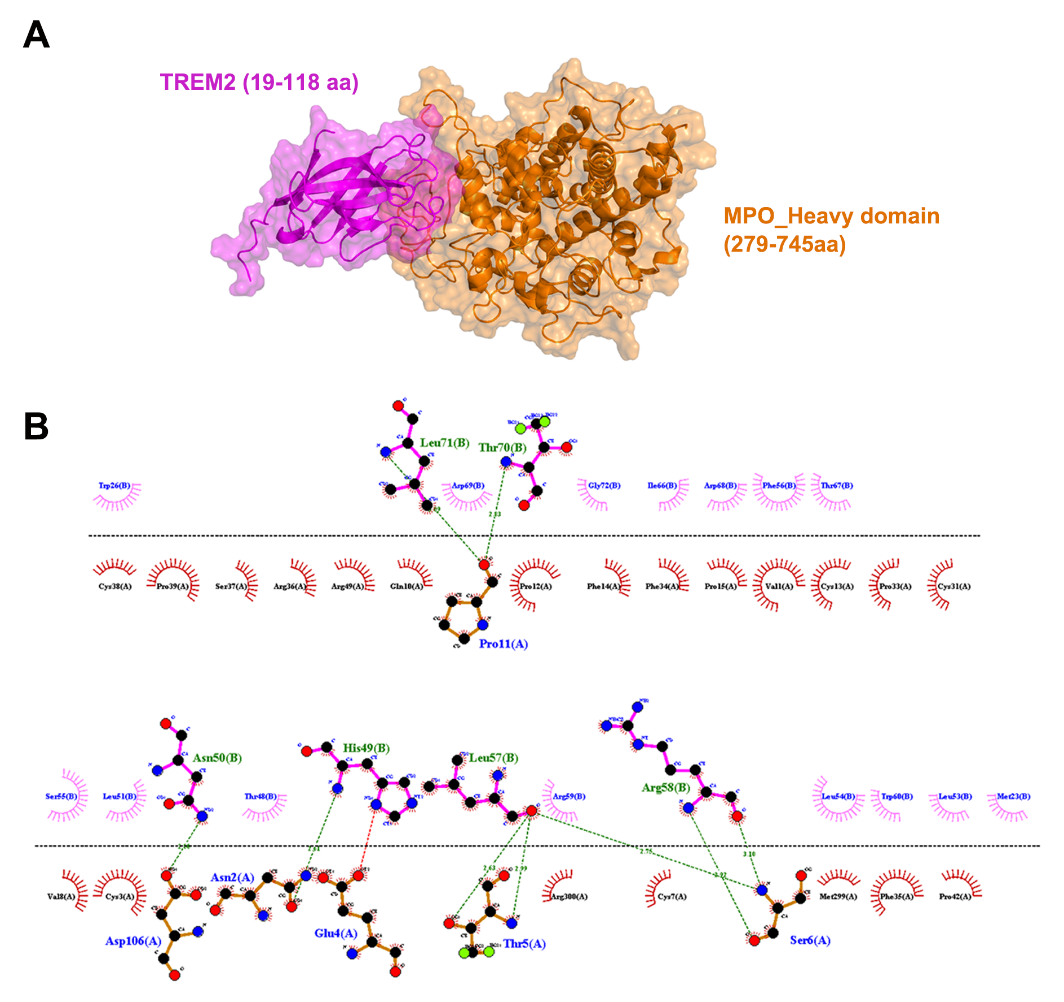


**Figure S11. Related to Figure 5. Structure prediction of the interaction between TREM2 (19-118aa) and MPO heavy domain (279-745 aa). (A)** Molecular docking analysis of the interaction between TREM2 (19-118 aa) and MPO heavy domain (279-745 aa). **(B)** Hydrogen bonding interactions were formed between TREM2 and the following residues of MPO: Asn2 (280 aa), Glu4 (282 aa), Thr5 (283 aa), Ser6 (284 aa), Pro11 (289 aa) and Asp106 (384 aa).


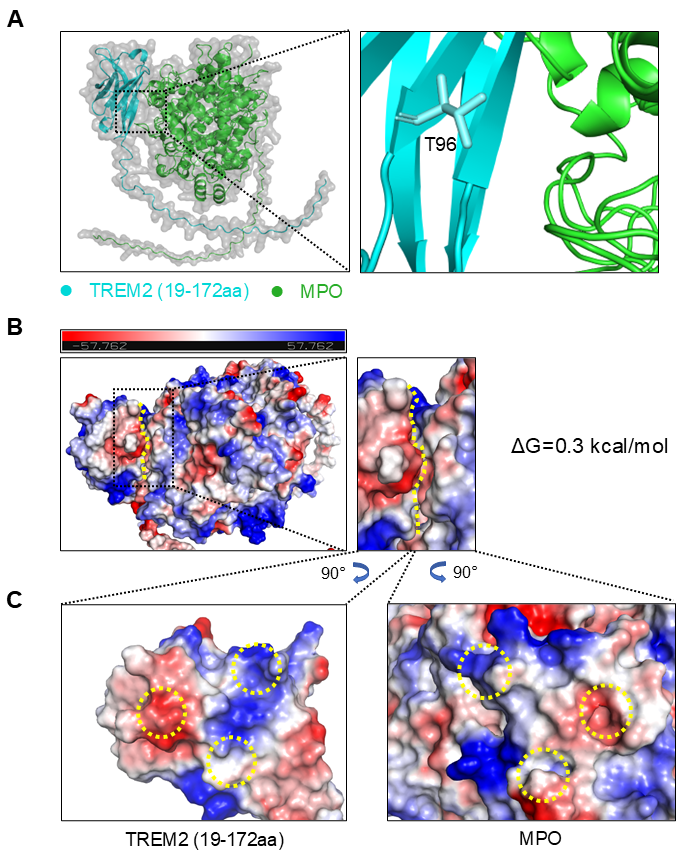


**Figure S12. related to Figure 5. Structure prediction of the interaction between TREM2 (WT) and MPO. (A)** The interaction between WT TREM2 (cyan) and MPO (green) was predicted by *AlphaFold v3.0* and presented in the mode of cartoon with semi-transparent surface (left side). The specific amino-acid residues forming bonds were shown in the stick mode (right side). **(B)** Charge distribution and potential in TREM2 (WT) and MPO structures after docking (left side). Red represents negative charge, and blue represents positive charge. The interaction area was magnified on the right side, which was depicted by yellow dashed line. ΔG indicates the binding free energy between two proteins. **(C)** The interaction surfaces located in TREM2 (WT) and MPO were presented after a horizontal flip of 90 degrees, respectively. The yellow dashed circles on TREM2 (WT) and MPO marked the position with strong interaction.


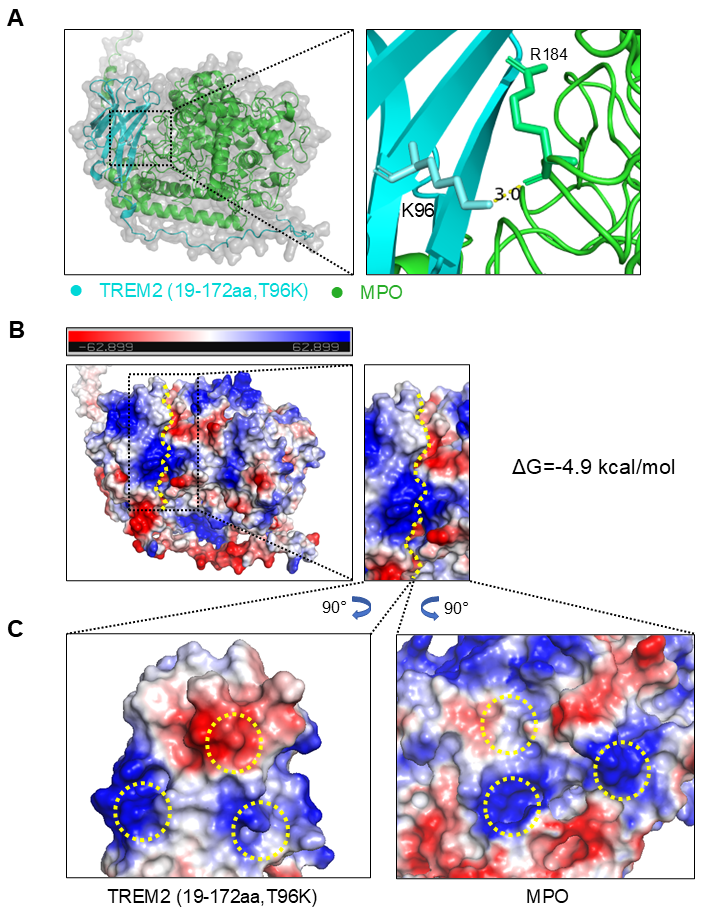


**Figure S13. related to Figure 5. Structure prediction of the interaction between TREM2 (T96K, mutation) and MPO. (A)** The interaction between T96K TREM2 (cyan) and MPO (green) was predicted by *AlphaFold v3.0* and presented in the mode of cartoon with semi-transparent surface (left side). The specific amino-acid residues forming bonds were shown in the stick mode (right side). **(B)** Charge distribution and potential in TREM2 (T96K) and MPO structures after docking (left side). Red represents negative charge, and blue represents positive charge. The interaction area was magnified on the right side, which was depicted by yellow dashed line. ΔG indicates the binding free energy between two proteins. **(C)** The interaction surfaces located in TREM2 (T96K) and MPO were presented after a horizontal flip of 90 degrees, respectively. The yellow dashed circles on TREM2 (T96K) and MPO marked the position with strong interaction.


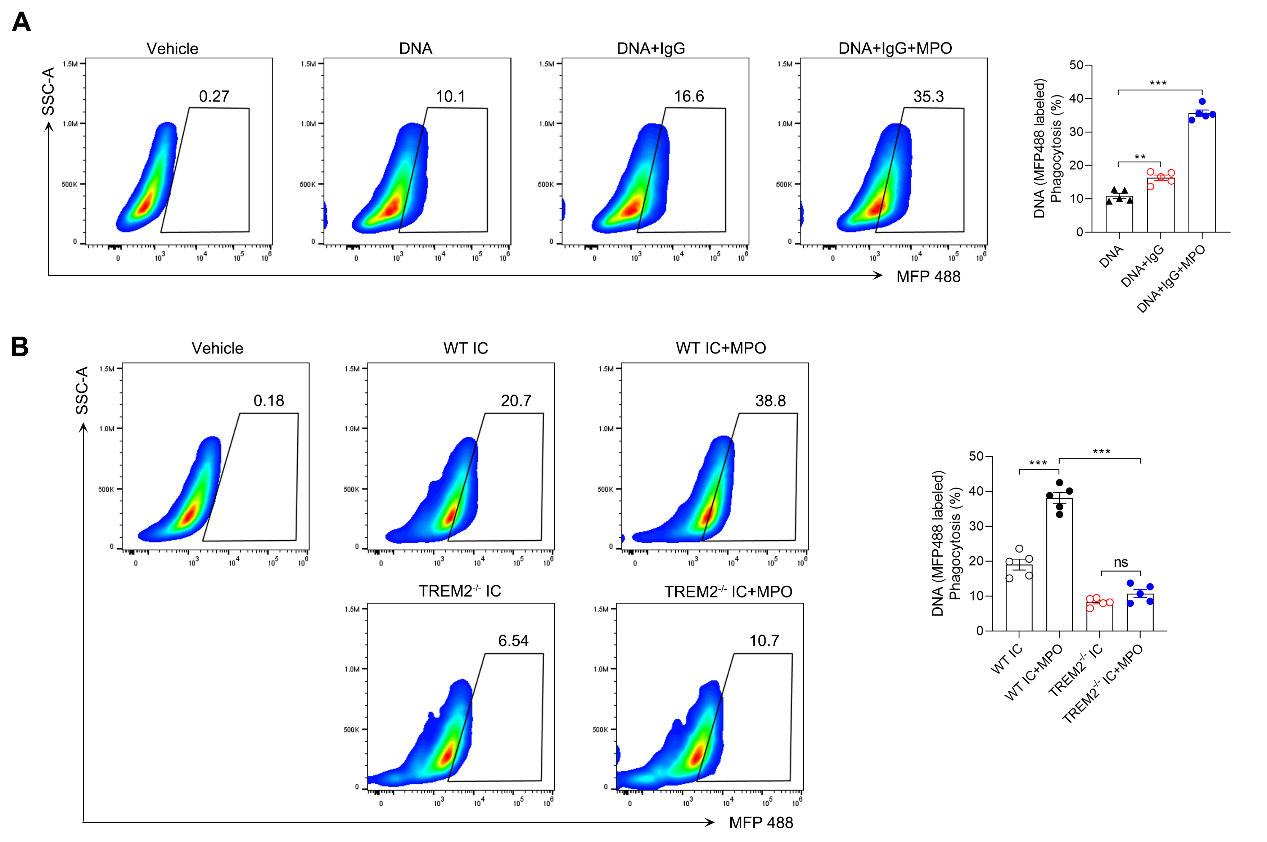


**Figure S14. related to Figure 5. MPO facilitates the uptake of DNA-containing ICs by BMDCs in a TREM2-dependent manner. (A)** The DNA was labeled with MFP488. WT BMDCs were cocultured with labeled DNA, IC (labeled DNA + IgG), or IC + MPO for 3 hours. The ability of WT BMDCs to phagocytose DNA was assessed by flow cytometry (n=5, One-way ANOVA). (B) WT or *Trem2*^-/-^ BMDCs were cocultured with IC (labeled DNA + IgG), or IC + MPO for 2 hours. The ability of WT or *Trem*2^-/-^ BMDCs to phagocytose DNA-containing IC was assessed by flow cytometry (n=5, One-way ANOVA). The results are presented as the mean ± SEM from three separate experiments. *, P < 0.05; **, P < 0.01; ***, P < 0.001; ns, no significance difference.


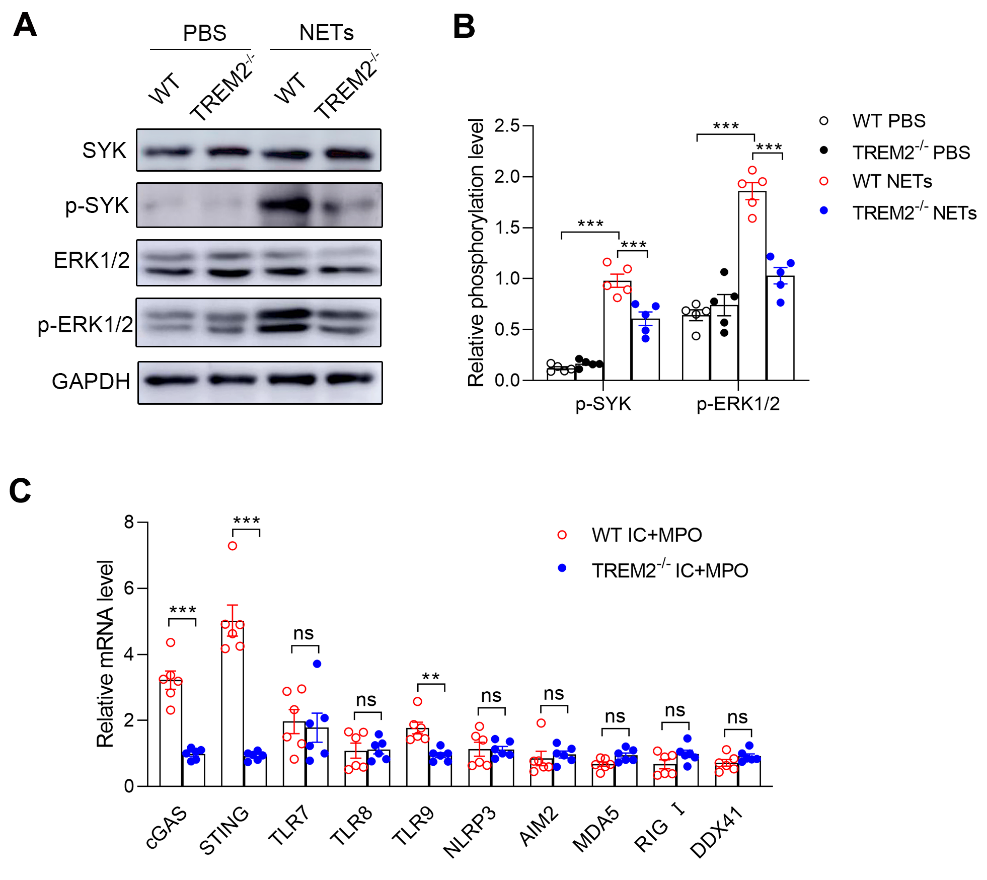


**Figure S15. related to Figure 6. TREM2 mediates NETs/MPO-induced SYK/ERK1/2 and cGAS/STING activation (A)** WT and *Trem2*^-/-^ BMDCs were stimulated with NETs for 24 hours. The protein levels of total SYK, ERK1/2, along with their phosphorylated forms were determined by WB. **(B)** The band intensities of p-SYK, p-ERK1/2were quantified using ImageJ software and normalized to their corresponding total protein levels (n=5, one-way ANOVA). **(C)** WT and *Trem*2^-/-^ BMDCs were stimulated with IC+MPO for 24 hours. The mRNA level of pattern recognition receptors (cGAS, STING, TLR7, TLR8, TLR9, NLRP3, AIM2, MDAD, RIG Ⅰ, DDX41) were detected by RT-PCR (n=6, unpaired 2-tailed Student’s t test). The results are presented as the mean ± SEM from three independent experiments. ns indicates no significance, *P < 0.05, **P < 0.01, ***P < 0.001.


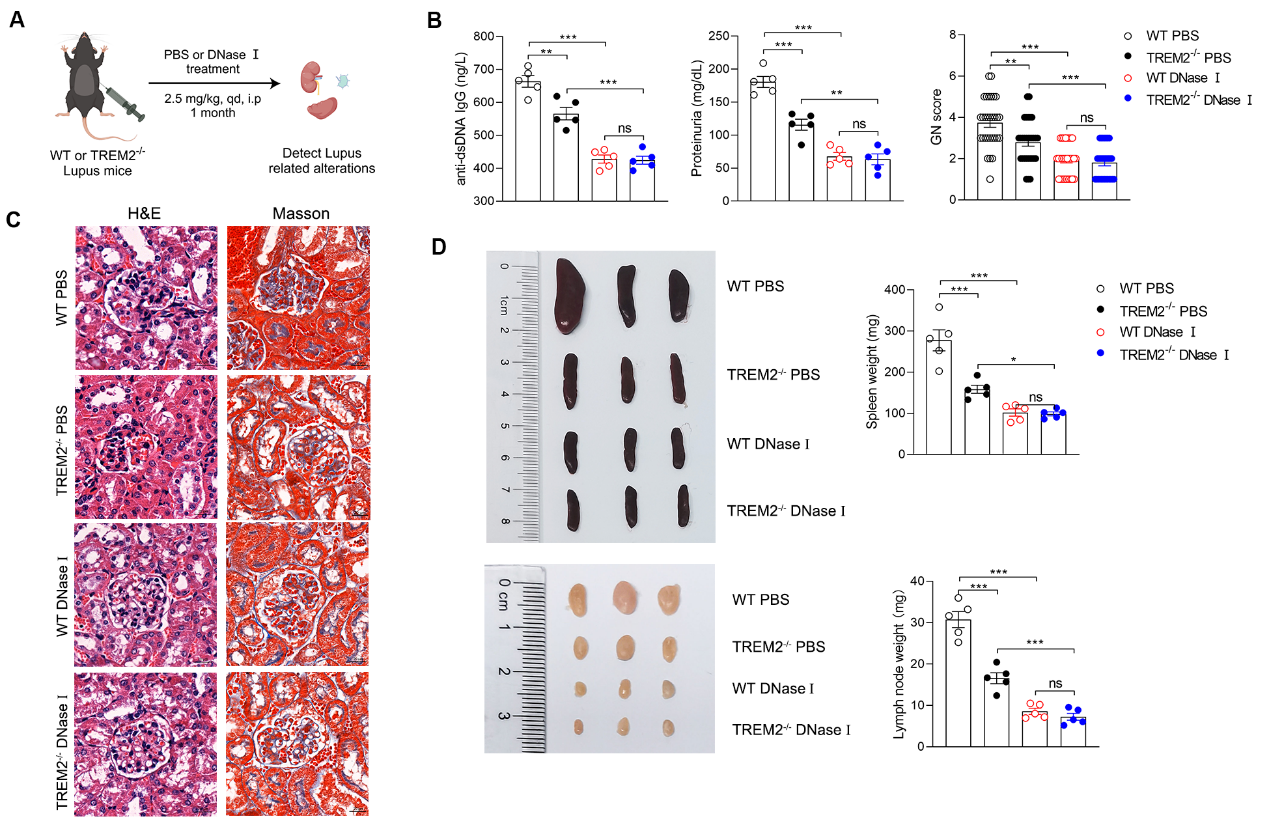


**Figure S16. related to Figure 7. Elimination of NETs protect against TREM2-driven lupus progression. (A)** WT or *Trem2*^-/-^ lupus mice were administered with DNase I or PBS by intraperitoneal injection (i.p.) every day for one month. **(B)** The serum and urine samples from each mouse of the indicated groups were collected after DNase I or PBS treatment for one month. The anti-dsDNA Abs and proteinuria levels were detected by ELISA (n=5, one-way ANOVA). **(B-C)** The paraffin-embedded kidney sections were stained with H&E and Masson. The Kidney pathology was evaluated by GN scores (n=30, one-way ANOVA). Scale bar, 20 μm. **(D)** The gross appearance and the weight of spleens or lymph nodes from mice of the indicated groups were recorded (n=5, one-way ANOVA). The results are presented as the mean ± SEM from three separate experiments. *, P < 0.05; **, P < 0.01; ***, P < 0.001; ns, no significance difference.

**Table S1. Clinical and laboratory characteristics**

| **Characteristic** | **Healthy controls (n=30)** | **SLE patients (n=46)** |
| --- | --- | --- |
| Sex (male/female) | 5/25 | 7/39 |
| Age (mean±SEM) | 34.33±2.44 | 35.80±1.67 |
| SLEDAI-2K scores | - | 5.02±0.49 |
| Anti-dsDNA Abs (IU/mL) | - | 227.99±29.70 |
| Proteinuria (mg/d) | - | 1085.27±264.19 (n=38) |
| C3 (mg/dl) | - | 67.98±4.67 |
| C4 (mg/dl) | - | 16.00±0.96 |
| Anti-ANA  (Positive/negative) | - | 46/0 |

**Table S2. The potential proteins of NETs interacting with TREM2**

| **Protein Accession** | **-10LogP** | **Gene Name** |
| --- | --- | --- |
| P05164-3\|PERM_HUMAN | 232.8472 | MPO |
| P20160\|CAP7_HUMAN | 212.3488 | AZU1 |
| O60814\|H2B1K_HUMAN | 190.8327 | H2BC12 |
| Q16778\|H2B2E_HUMAN | 190.8327 | H2BC21 |
| P58876\|H2B1D_HUMAN | 190.8327 | H2BC5 |
| Q5QNW6\|H2B2F_HUMAN | 190.8327 | H2BC18 |
| Q5QNW6-2\|H2B2F_HUMAN | 190.8327 | H2BC18 |
| Q99879\|H2B1M_HUMAN | 190.8327 | H2BC14 |
| Q99877\|H2B1N_HUMAN | 190.8327 | H2BC15 |
| P57053\|H2BFS_HUMAN | 190.8327 | H2BS1 |
| P62807\|H2B1C_HUMAN | 190.8327 | H2BC4 |
| Q93079\|H2B1H_HUMAN | 190.8327 | H2BC9 |
| P33778\|H2B1B_HUMAN | 190.8327 | H2BC3 |
| P06899\|H2B1J_HUMAN | 190.8327 | H2BC11 |
| P23527\|H2B1O_HUMAN | 190.8327 | H2BC17 |
| P08311\|CATG_HUMAN | 153.168 | CTSG |
| P59665\|DEF1_HUMAN | 89.89475 | DEFA1 |
| P02042\|HBD_HUMAN | 89.5775 | HBD |
| P62805\|H4_HUMAN | 69.09165 | H4C1 |
| P47929\|LEG7_HUMAN | 64.54755 | LGALS7 |
| P24158\|PRTN3_HUMAN | 50.07977 | PRTN3 |
| P13727-2\|PRG2_HUMAN | 49.57014 | PRG2 |
| P11171\|41_HUMAN | 49.06636 | EPB41 |
| P11171-7\|41_HUMAN | 49.06636 | EPB41 |

**Table S3. Primers for RT-PCR**

| **Gene name** | **Direction** | **Sequence 5’ to 3’** |
| --- | --- | --- |
| mIFNα | Forward | GGATGTGACCTTCCTCAGACTC |
|  | Reverse | ACCTTCTCCTGCGGGAATCCAA |
| mIFNβ | Forward | CGCTGCGTTCCTGCTGT |
|  | Reverse | CGCCCTGTAGGTGAGGTTGA |
| mBAFF | Forward | CAGCGACACGCCGACTATAC |
|  | Reverse | CCTCCAAGGCATTTCCTCTTTT |
| mIL-4 | Forward | CGTCTGTAGGGCTTCCAAGG |
|  | Reverse | AGGCATCGAAAAGCCCGAA |
| mIL-6 | Forward | TACCACTTCACAAGTCGGAGGC |
|  | Reverse | CTGCAAGTGCATCATCGTTGTTC |
| mIL-10 | Forward | TTTCAAACAAAGGACCAG |
|  | Reverse | GGATCATTTCCGATAAGG |
| mIL-12 | Forward | GGGGTGTAACCAGAAAGG |
|  | Reverse | CATGAGGAATTGTAATAGCG |
| mβ-actin | Forward | CATTGCTGACAGGATGCAGAAGG |
|  | Reverse | TGCTGGAAGGTGGACAGTGAGG |
